# Metabolic engineering of *Escherichia coli* for de novo production of 3-phenylpropanol via retrobiosynthesis approach

**DOI:** 10.1186/s12934-021-01615-1

**Published:** 2021-06-27

**Authors:** Zhenning Liu, Xue Zhang, Dengwei Lei, Bin Qiao, Guang-Rong Zhao

**Affiliations:** 1 Frontier Science Center for Synthetic Biology and Key Laboratory of Systems Bioengineering (Ministry of Education), School of Chemical Engineering and Technology, Tianjin University, Yaguan Road 135, Jinnan District, Tianjin, 300350 China; 2 Georgia Tech Shenzhen Institute, Tianjin University, Tangxing Road 133, Nanshan District, Shenzhen, 518071 China

**Keywords:** 3-Phenylpropanol, Synthetic biology, Retrobiosynthesis, Metabolic engineering, *Escherichia coli*

## Abstract

**Background:**

3-Phenylpropanol with a pleasant odor is widely used in foods, beverages and cosmetics as a fragrance ingredient. It also acts as the precursor and reactant in pharmaceutical and chemical industries. Currently, petroleum-based manufacturing processes of 3-phenypropanol is environmentally unfriendly and unsustainable. In this study, we aim to engineer *Escherichia coli* as microbial cell factory for de novo production of 3-phenypropanol via retrobiosynthesis approach.

**Results:**

Aided by in silico retrobiosynthesis analysis, we designed a novel 3-phenylpropanol biosynthetic pathway extending from l-phenylalanine and comprising the phenylalanine ammonia lyase (PAL), enoate reductase (ER), aryl carboxylic acid reductase (CAR) and phosphopantetheinyl transferase (PPTase). We screened the enzymes from plants and microorganisms and reconstructed the artificial pathway for conversion of 3-phenylpropanol from l-phenylalanine. Then we conducted chromosome engineering to increase the supply of precursor l-phenylalanine and combined the upstream l-phenylalanine pathway and downstream 3-phenylpropanol pathway. Finally, we regulated the metabolic pathway strength and optimized fermentation conditions. As a consequence, metabolically engineered *E. coli* strain produced 847.97 mg/L of 3-phenypropanol at 24 h using glucose-glycerol mixture as co-carbon source.

**Conclusions:**

We successfully developed an artificial 3-phenylpropanol pathway based on retrobiosynthesis approach, and highest titer of 3-phenylpropanol was achieved in *E. coli* via systems metabolic engineering strategies including enzyme sources variety, chromosome engineering, metabolic strength balancing and fermentation optimization. This work provides an engineered strain with industrial potential for production of 3-phenylpropanol, and the strategies applied here could be practical for bioengineers to design and reconstruct the microbial cell factory for high valuable chemicals.

**Supplementary Information:**

The online version contains supplementary material available at 10.1186/s12934-021-01615-1.

## Introduction

3-Phenylpropanol, which gives a pleasant hyacinth-mignonette odor and an apricot-like taste, has been extensively used in foods, beverages and cosmetics as a fragrance ingredient by approval of the United States Food and Drug Administration (21 CFR 172.515) [1]. 3-Phenylpropanol is also the substrate for the production of a fragrance ingredient 3-phenylpropyl acetate [2], and a pharmaceutical phenprobamate, which is a central skeletal muscle relaxant for treatment of muscle cramps and spasticity [3, 4]. Moreover, 3-phenylpropanol acts as the reactant for the synthesis of amines, ethers, and other chemicals with applications in coatings, resins and pharmaceutical building blocks [5, 6]. Just for uses in fragrance industry, the global consumption of 3-phenylpropanol was estimated to range between 100 and 1000 metric tons per annum [7]. Currently, the manufacturing process for the production of 3-phenylpropanol is petroleum-based, commonly relying on the hydrogenation of cinnamaldehyde in the presence of metal catalysts [8, 9].

**Fig. 1:**
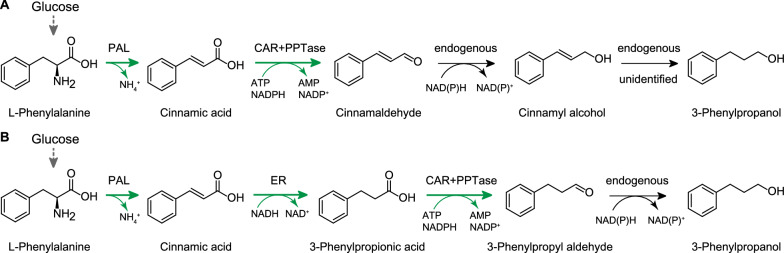
The 3-phenylpropanol biosynthetic pathways. **A** The proposed pathway in *S. cerevisiae* [23, 24]. **B** The retrosynthetically designed artificial pathway in *E. coli* in this study. Green arrow indicated the heterologous enzymes: *PAL* l-phenylalanine ammonia lyase, *CAR* carboxylic acid reductase, *PPTase* phosphophantethinyl transferase, *ER* enoate reductase

As an eco-friendly and economic approach, engineering microorganisms has become an attractive alternative to efficiently produce high-value compounds, such as flavors, fragrances, cosmetics, pharmaceuticals, solvents, biofuels and other chemicals [10–22]. Bioproduction of 3-phenylpropanol has been reported in *Saccharomyces cerevisiae* [23]*.* By introducing phenylalanine ammonia lyase (PAL) gene from *Photorhabdus luminescens*, aryl carboxylic acid reductase (CAR) gene from *Nocardia *sp., and phosphopantetheinyl transferase (PPTase) gene from *Escherichia coli*, engineered *Saccharomyces cerevisiae* produced 212.9 mg/L of 3-phenylpropanol from glucose [23, 24] (Fig. 1A). However, using the whole lyophilised *E. coli* cell extract expressing these three heterologous enzymes, the in vitro enzymatic reaction with substrate l-phenylalanine revealed that the major product was cinnamyl alcohol and 3-phenylpropanol was the byproduct [25]. It was likely that the enzymatic properties of the unknown endogenous enzymes involved in the 3-phenylpropanol biosynthetic pathway between *S. cerevisiae* and *E. coli* were seriously different, which hindered further improvement of 3-phenylpropanol production in microorganisms. Retrobiosynthesis is an approach for biosynthetic pathway design from target molecules to cellular metabolites, using the biotransformation rules that present the rearrangement of atoms and bonds in enzymatic reactions [26, 27]. With great potential in synthetic biology and metabolic engineering, retrobiosynthesis has been applied in the production of didanosine [28], 5‑aminolevulinic acid [29], and short-chain primary amines [30]. Furthermore, the current development of retrobiosynthesis tools for biosynthesis of valuable chemicals [27, 31, 32] provides a more feasible approach to rationally design a novel artificial pathway according to the available enzymatic reactions from databases, and then implement systems metabolic engineering strategies for efficient production of 3-phenylpropanol in microbes.

In this study, using the retrobiosynthesis analysis strategy, we designed a novel biosynthetic pathway and engineered an recombinant *E. coli* strain capable of de novo production of 3-phenylpropanol from glucose (Fig. 1B). First, we conducted in silico retrobiosynthesis of 3-phenylpropanol aided by RetroPath 2.0 software, and identified a candidate pathway from enumerated pathways. This pathway extended from l-phenylalanine and consisted of PAL, enoate reductase (ER), CAR and PPTase, which was different from previous reported pathway in yeast. Next, we refactored the designed pathway in *E. coli* by expressing the heterologous genes from different species. We constructed the de novo producing strain by chromosome engineering and tuned the expression pattern of the pathway genes. After the optimization of culture conditions, the engineered *E. coli* produced 847.97 mg/L of 3-phenylpropanol from glucose in 24 h of flask-shake fermentation, which is the highest titer achieved in microbial production of 3-phenylpropanol up to date.

## Results and discussion

### Retrobiosynthetic design of 3-phenylpropanol biosynthetic pathway

**Fig. 2:**
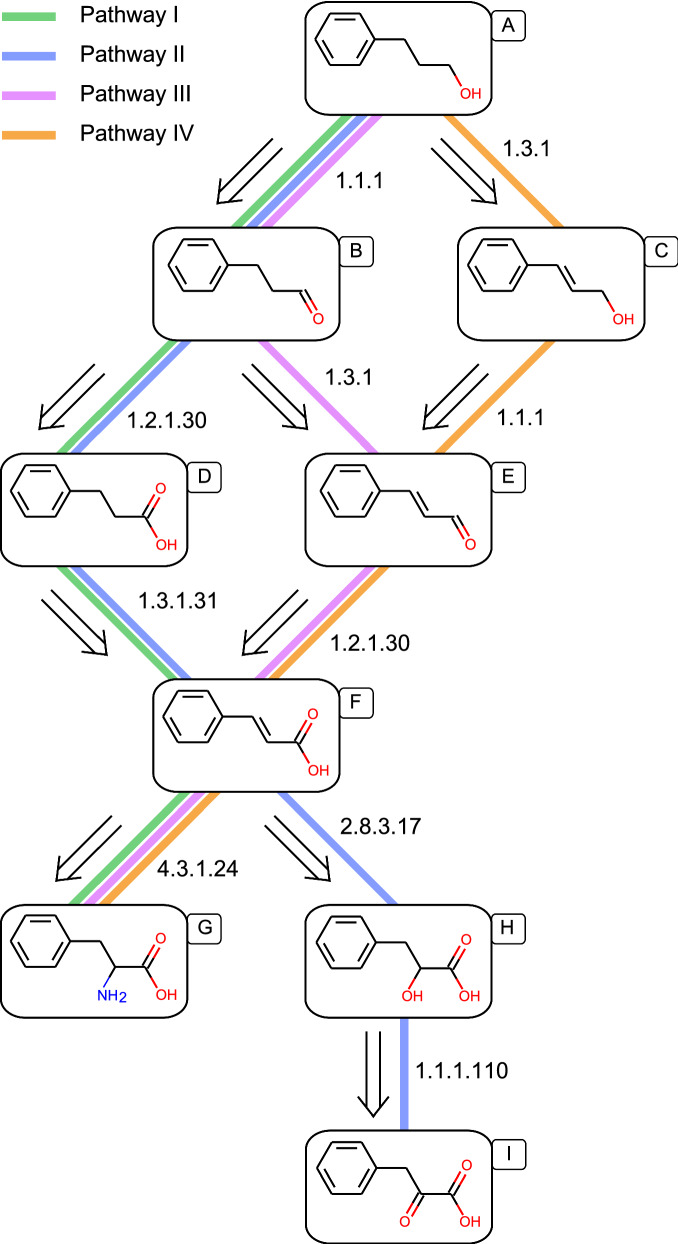
Enumeration of 3-phenylpropanol biosynthetic pathways. All of the four pathways were extending from l-phenylalanine and phenylpyruvate in *E. coli* native l-phenylalanine metabolism and depicted by RetroPath 2.0 [33]. Pathway I and II was automatically generated by retrosynthesis workflow of RetroPath 2.0 based on reactions from databases. Pathway III and IV were functional pathways in previous reports [23–25]. The reactions in pathways were represented by EC numbers. The compound names are as follows: 3-phenylpropanol (**A**), 3-phenylpropyl aldehyde (**B**), cinnamyl alcohol (**C**), 3-phenylpropionic acid (**D**), cinnamaldehyde (**E**), cinnamic acid (**F**), l-phenylalanine (**G**), 3-phenyllactic acid (**H**), phenylpyruvate (**I**)

Due to the lack of characterization for the natural pathway in plants and limited knowledge on established pathway for 3-phenylpropanol biosynthesis, we aimed to enumerate possible 3-phenylpropanol pathways from a retrobiosynthesis viewpoint. Using RetroPath 2.0 [33], an automated retrosynthesis workflow, we predicted potential pathways based on enormous amount of generative reactions in public databases [34], and two potential pathways were generated extending from the native l-phenylalanine metabolism in *E. coli* (Fig. 2, pathways I and II). We manually added two functional pathways (pathway III and IV) which were proposed previously in *E. coli* [25] and yeast [23, 24], respectively, and four pathways were totally depicted.

As shown in Fig. 2, except for longer pathway II which underwent five steps, the other three pathways had the same length with similar reaction types. The α-hydroxyl functionality can be obtained from an α-aldehyde group catalyzed by *E. coli* endogenous aldo–keto reductases (AKRs, EC 1.1.1.346) or alcohol dehydrogenases (ADHs, EC 1.1.1.2) [35]. The difference among pathways I, III and IV was the order of the C=C double bond hydrogenation and the carboxyl group reduction of cinnamic acid. Most of ERs work well at anaerobic environment and are not suitable for aerobic fermentation [36]. Recently, the CaER from *Clostridium acetobutylicum* showed the oxygen tolerance in conversion of cinnamic acid to 3-phenylpropionic acid in *E. coli* [37]. The substrates of CAR enzymes include both saturated or unsaturated acids, ensuring the selective reduction of carboxyl group [38]. Thus, pathway I was selected as the candidate one among four proposed pathways with confirmed enzymes for each step.

To validate the feasibility of the retrobiosynthetically designed pathway extending *E. coli* native l-phenylalanine metabolism for de novo production of 3-phenylpropanol, we divided the full pathway into the upstream pathway for l-phenylalanine biosynthesis from glucose and the downstream pathway for the 3-phenylpropanol biosynthesis from L-phenylalanine. These two pathways were retro-synthetically reconstructed and optimized for de novo production of 3-phenylpropanol in the following experiments.

### Reconstructing the downstream pathway for 3-phenylpropanol biosynthesis from l-phenylalanine

**Fig. 3:**
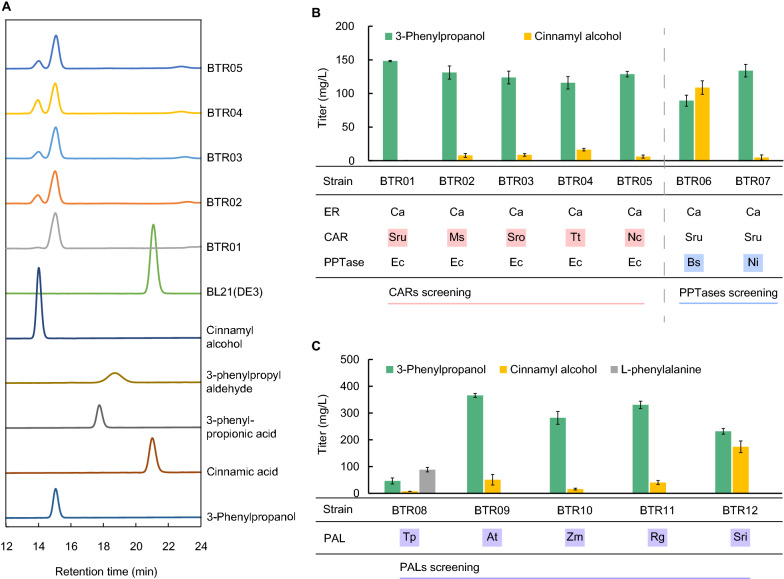
Reconstruction of the downstream pathway of 3-phenylpropanol biosynthesis from l-phenylalanine. **A** HPLC spectra of the culture supernatants of strains BTR01, BTR02, BTR03, BTR04 and BTR05, which harbored CaER, EcPPTase and various CARs. **B** Performance of strains harboring various CARs and PPTases. 200 mg/L of cinnamic acid was supplemented in the medium. **C** Performance of strains with various PALs. 500 mg/L of l-phenylalanine and 0.1 mM IPTG were supplemented in the medium. All samples were collected and analyzed at 48 h. Source organisms of pathway enzymes are abbreviated: Ca, *Clostridium acetobutylicum*; Ec, *Escherichia coli*; Sru, *Segniliparus rugosus*; Ms, *Mycobacterium smegmatis*; Sro, *Segniliparus rotundus*; Tt, *Thermothelomyces thermophile*; Nc, *Neurospora crassa*; Bs, *Bacillus subtilis*; Ni, *Nocardia iowensis*; Tp, *Trifolium pratense*; At, *Arabidopsis thaliana*; Zm, *Zea mays*; Rg, *Rhodotorula glutinis*; Sri, *Streptomyces rimosus*

In our designed downstream pathway which comprised PAL, ER, CAR/PPTase, and endogenous alcohol dehydrogenases (ADHs) or aldo–keto reductases (AKRs), except for the previously reported CaER from *Clostridium acetobutylicum* which was suitable for the conversion of 3-phenylpropionic acid from cinnamic acid under aerobic conditions [37], the other suitable enzymes remain to be evaluated. The reductive reaction of carboxylic acid moiety of 3-phenylpropionic acid catalyzed by post-translationally PPTase-activated CAR is crucial for 3-phenylpropanol biosynthesis and five CARs from different species were chosen as candidates: SruCAR from *Segniliparus rugosus* [25], MsCAR from *Mycobacterium smegmatis* [39], SroCAR from *Segniliparus rotundus* [40], TtCAR from *Thermothelomyces thermophila* [41], and NcCAR from *Neurospora crassa* [42]. We predicted that the unknown endogenous ADHs or AKRs could catalyze the conversion of 3-phenylpropanol from 3-phenylpropyl aldehyde. Thus, we screened the CAR and PPTase sequentially. Firstly, we cloned five candidate *CAR* genes into pETDuet-1, respectively, and transformed them together with the plasmid expressing the *CaER* gene and the *EcPPTase* gene of *E. coli* [41] in pCDFDuet-1, into *E. coli* BL21(DE3) to construct strains BTR01, BTR02, BTR03, BTR04 and BTR05. The fermentation was performed with supplementation of cinnamic acid, and the production of 3-phenylpropanol was analyzed by HPLC. Expectedly, as shown in Fig. 3A, five strains produced 3-phenylpropanol which has the same retention time as 3-phenylpropanol standard. The identity of 3-phenylpropanol was further confirmed by gas chromatography–mass spectrometry analysis (Additional file 1: Figure S1). The results indicated that the endogenous ADHs or AKRs actively worked as previous reports [13, 43] and that the ER, CAR and PPTase were essential for biosynthesis of 3-phenylpropanol when they were expressed in *E. coli*. As shown in Fig. 3B, compared to strain BTR01 which produced 148.34 mg/L of 3-phenylpropanol, strains BTR02, BTR03, BTR04 and BTR05 expressing different *CARs* genes produced less amounts of 3-phenylpropanol, ranging from 115.92 to 131.23 mg/L, indicating SruCAR was superior to the others in the biosynthetic pathway of 3-phenylpropanol from cinnamic acid. The minor amount accumulation of byproduct cinnamyl alcohol was probably caused by the promiscuity of CAR which catalyzed the reduction of carboxylic acid moiety of cinnamic acid into the formation of cinnamyl aldehyde followed by the endogenous reduction or dehydrogenation as previous report [25].

Considering the multiple functionality of PPTases involved in modification and regulation on activities and regeneration of polyketide synthases [44], we next investigated BsPPTase from *Bacillus subtilis* [45] which was commonly employed in the heterologous production of polyketides in *E. coli* and yeast [46], and NiPPTase from *Nocardia iowensis* [40] which was frequently used for activation of heterologous CARs [38]. As shown in Fig. 3B, BTR06 expressing BsPPTase and BTR07 expressing NiPPTase produced 89.30 mg/L and 133.99 mg/L of 3-phenylpropanol, respectively. NiPPase seemed nearly comparable to EcPPTase for production of 3-phenylpropanol, while BsPPTase was quite incompetent to do the work as large amount of cinnamyl alcohol was accumulated, indicating the incompatibility between BsPPTase and SruCAR, or the unknown effects of BsPPTase on the metabolic pathway. Taken together, we confirmed the novel biosynthetic pathway of 3-phenylpropanol composed of CaER, SruCAR and EcPPTase, which can efficiently catalyze the formation of 3-phenylpropanol from cinnamic acid in *E. coli*, with no accumulation of the precursor cinnamic acid and byproduct cinnamyl alcohol in strain BTR01.

PAL enzyme links 3-phenylpropanol biosynthetic pathway to *E. coli* endogenous metabolite l-phenylalanine by converting l-phenylalanine to cinnamic acid (Fig. 1B). In order to eliminate the bottleneck at this metabolic node, we introduced different PALs into the designed pathway and investigated their effects on 3-phenylpropanol production. Three identified plant PALs (TpPAL1 [47], AtPAL2 [48], ZmPAL2 [49]) and one characterized *Rhodotorula* TAL (RgTAL[37]), together with one putative PAL from *Streptomyces rimosus* [50] were investigated. We cloned candidate *PALs* genes or TAL gene into pRSFDuet-1 and transformed them into strain BTR01 to obtain strains BTR08, BTR09, BTR10, BTR11 and BTR12 (Fig. 3C), respectively. We added l-phenylalanine in the cultivation medium, and measured the metabolites after fermentation. As shown in Fig. 3C, all resulting strains harboring tested PALs or TAL produced 3-phenylpropanol from precursor l-phenylalanine. However, the contribution of different PALs to the formation of 3-phenylpropanol remarkably varied. Strain BTR08 showed the lowest titer of 3-phenylpropanol (46.20 mg/L), while strains BTR10, BTR11 and BTR12 produced large amounts of 3-phenylpropanol, 281.90 mg/L, 330.36 mg/L and 231.66 mg/L, respectively. Strain BTR09 produced highest 3-phenylpropanol at the titer of 365.59 mg/L, 6.9-fold higher than that by strain BTR08, without the accumulation of cinnamic acid, indicating that AtPAL2 from *A. thaliana* was the most efficient and suitable in the designed biosynthetic pathway.

By experimental production of 3-phenylpropanol from l-phenylalanine in *E. coli*, we demonstrated the feasibility and high performance of designed pathway I. For pathway III in previous study [25], the low activities of *E. coli* endogenous C=C double-bond reductases might lead to the formation of cinnamyl alcohol instead of 3-phenylpropanol as the major product. As for pathway IV in yeast, the endogenous reductases might be more active than those in *E. coli*, and more 3-phenylpropanol was produced [23, 24]. Unidentified endogenous C=C double-bond reductases and the redox status might differ between *E. coli* cells and yeast cells, which resulted in different final products. CaER is an excellent reductase and could efficiently convert cinnamic acid to 3-phenylpropionic acid under aerobic conditions [37]. When the CaER was introduced to construct pathway I, the metabolic limitation at the C=C double-bond reduction node in 3-phenylpropanol biosynthetic pathway was removed, and 3-phenylpropanol was achieved as the major product in *E. coli* (Fig. 3C). Thus, we refactored the downstream pathway for 3-phenylpropanol biosynthesis which comprised AtPAL2, CaER, SruCAR, and EcPPTase, and attempted to optimize the upstream pathway in *E. coli* in following study.

### Chromosome engineering of upstream pathway for the de novo biosynthesis of 3-phenylpropanol

**Fig. 4:**
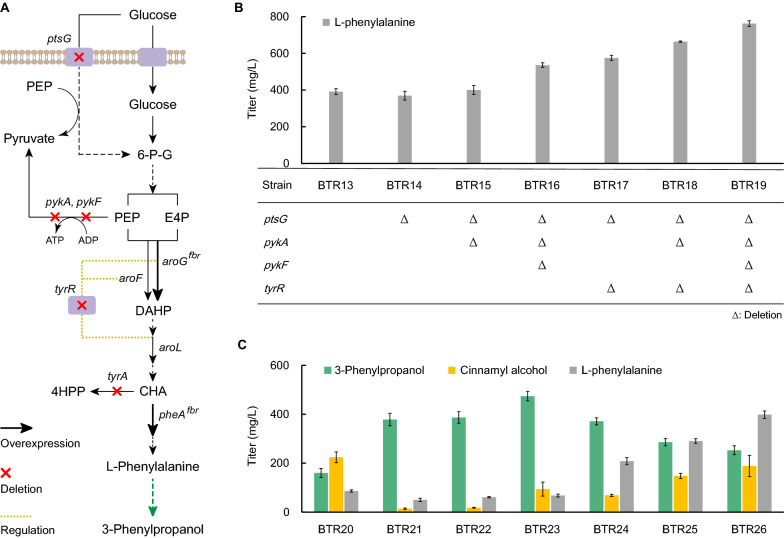
Compatible combination of upstream l-phenylalanine pathway and downstream 3-phenylpropanol pathway via chromosome engineering. **A** Overview of the upstream l-phenylalanine pathway in *E. coli*. **B** Enhancing the upstream pathway for l-phenylalanine biosynthesis from glucose by combinatorial chromosome engineering. Production of L-phenylalanine was detected after 36 h of fermentation. **C** Compatibility examination of the designed downstream pathway combining with the various modified upstream l-phenylalanine pathways for de novo production of 3-phenylpropanol from 10 g/L glucose. The inducer IPTG was added at a concentration of 0.01 mM. The performance was evaluated after 30 h of fermentation. Abbreviations: *6-P-G* 6-phosphate d-glucose, *PEP* phosphoenolpyruvate, *E4P* erythrose 4-phosphate, *DAHP* 3-deoxy-arabino-heptulonate 7-phosphate, *CHA* chorismic acid, *4HPP* 4-hydroxyphenylpyruvate, *fbr* feedback inhibition resistance

In order to achieve the de novo biosynthesis of 3-phenylpropanol, we used l-phenylalanine overproducing chassis BWH18 from our previous work [51] to enlarge the metabolic flux to upstream l-phenylalanine pathway from glucose by chromosome engineering. Since strain BWH18 was derived from *E. coli* BW25113 by integrating the *aroG*^fbr^-*pheA*^fbr^ genes and deleting the *tyrA* gene to relieve the feedback and competitive inhibitions, we integrated T7 RNA polymerase gene in BWH18 chromosome to confer 3-phenylpropanol biosynthetic genes of the downstream pathway to be controlled under T7 promoter, generating strain BTR13. Four potential targets can be disrupted to enhance the metabolic flux to l-phenylalanine from glucose in *E. coli* (Fig. 4A). Phosphoenolpyruvate (PEP) is one precursor for l-phenylalanine biosynthesis, and the PEP-dependent phosphotransferase system (PTS) is a major system for glucose transport, in which nearly 50% of PEP was consumed as the phosphate donor [52]. Thus we deleted the *ptsG* gene of strain BTR13 to construct strain BTR14, and the titer of l-phenylalanine was not obviously changed (Fig. 4B). PEP is also consumed and converted to pyruvate in glycolysis [53]. In order to conserve PEP, we deleted the *pykA* and *pykF* genes encoding pyruvate kinases, and the triple deletion strain BTR16 produced 535.87 mg/L of l-phenylalanine, a 36.90% increase than strain BTR13, in consistent with previous reports [54, 55]. l-Phenylalanine biosynthesis is negatively regulated by the repressor TyrR targeting the transcriptional expression of the *aroG* and *aroF* genes encoding 3-deoxy-arabino-heptulonate 7-phosphate (DAHP) synthase isoenzymes and the *aroL* gene encoding shikimate kinase [56]. We deleted the *tyrR* gene in combination with disruption of the *ptsG* and/or *pyk* genes to construct strains BTR17, BTR18 and BTR19. Compared to strain BTR13, the combinatorial effects of gene disruption enabled 47.00%, 69.68% and 94.73% improvement of l-phenylalanine production in strains BTR17, BTR18 and BTR19, respectively. The quadruple deletion strain BTR19 exhibited the highest production of l-phenylalanine with a titer of 762.25 mg/L.

We then introduced the downstream 3-phenylpropanol pathway to each of aforementioned l-phenylalanine overproducing strains, and examined the compatibility of the downstream pathway with various upstream pathways by comparing the productive performance of engineered strains. As shown in Fig. 4C, strain BTR20 produced 160.05 mg/L of 3-phenylpropanol, and a large amount of byproduct cinnamyl alcohol (224.03 mg/L) was accumulated, indicating that the chassis strain BTR13 was not fit for producing 3-phenylpropanol. When the *ptsG* and/or *pyk* genes were deleted, the byproduct faded significantly, and the production of 3-phenylpropanol was greatly increased in strains BTR21-BTR23, of which, strain BTR23 with the triple deletion of the *ptsG, pykF* and *pykA* genes gave the highest production of 3-phenylpropanol with the titer of 473.75 mg/L, 2.0-fold higher than BTR20.

Although deleting the *tyrR* gene benefited the biosynthesis of l-phenylalanine in strains BTR17, BTR18, and BTR19, both the precursor l-phenylalanine and byproduct cinnamyl alcohol were seriously accumulated in strains BTR24, BTR25, and BTR26, and the production of 3-phenylpropanol was not improved as the same as that of l-phenylalanine in the chassis strains, revealing the incompatibility between the artificial biosynthetic pathway of 3-phenylpropanol and the physiological status of chassis strains of BTR24, BTR25, and BTR26. It was speculated that the metabolic flux to the upstream pathway for l-phenylalanine biosynthesis exceeded that to the downstream pathway for 3-phenylpropanol biosynthesis. In addition to genes involved in l-phenylalanine metabolism, the TyrR regulon consists of a diverse range of members awaiting to be identified [57, 58], which might indirectly influence 3-phenylpropanol biosynthesis.

### Balancing the metabolic strength of the downstream 3-phenylpropanol pathway

**Fig. 5:**
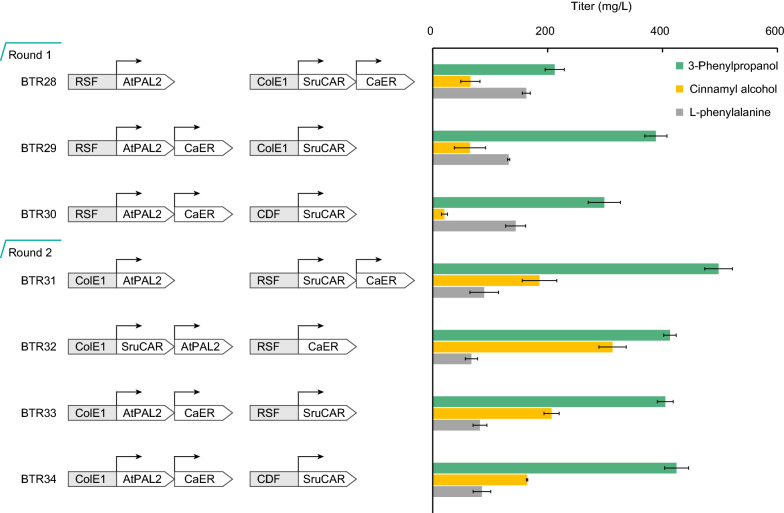
Balancing metabolic strength of the downstream 3-phenylpropanol pathway. Various expression patterns were designed by expressing the *AtPAL2*, *CaER* and *SruCAR* genes at both the high (RSF ori) and middle (ColE1 ori) or low (CDF ori) copy number. The fermentation was conducted in the presence of 10 g/L glucose and 0.01 mM IPTG, and the broth was analyzed after 48 h of cultivation

Based on the auxiliary function of EcPPTase for activation of SruCAR, we proposed that high overexpression of EcPPTase would be unnecessary. The gene encoding EcPPTase was removed from the expression plasmid and integrated in the chromosome of engineered *E. coli* strain. As expectedly, the resulting strain BEL09 produced similar amount of 3-phenylpropanol (461.25 mg/L) to strain BTR23 (473.75 mg/L). To alleviate the metabolic burden caused by three expression plasmids, we regulated the metabolic strength of the *AtPAL2*, *CaER* and *SruCAR* genes using two compatible plasmids derived from pRSFDuet-1 (high copy number, RSF ori), pETDuet-1 (middle copy number, ColE1 ori) or pCDFDuet-1 (low copy number, CDF ori) (Fig. 5). We did two rounds of regulation tests. In the first round of test, compared to strain BTR28, coexpressing the *AtPAL2* and *CaER* genes in pRSFDuet-1 increased the production of 3-phenylpropanol, whenever the *SruCAR* gene was expressed in pETDuet-1 or pCDFDuet-1. Since l-phenylalanine precursor was accumulated in tested strains of the first round, the second round of test was carried out by down-regulating expression of the *AtPAL2* gene with or without the *CaER* or *SruCAR* genes in ColE1-originated middle copy number plasmid pETDuet-1. Compared to the strains tested in the first round, accumulation of l-phenylalanine was decreased, while concentration of cinnamyl alcohol was increased in strains BTR31-BTR34. Among them, strain BTR31 showed the best performance on fermentation, and produced 497.49 mg/L of 3-phenylpropanol, 1.3-fold higher amount than that of strain BTR28. The results indicated the expression of both *CaER* and *SruCAR* genes in pRSFDuet-1 was beneficial for higher production of 3-phenylpropanol. We tried to improve the production titer and reduce the accumulation of byproduct by optimizing fermentation conditions in next section.

### Optimizing fermentation conditions for further improvement of 3-phenylpropanol production

**Fig. 6:**
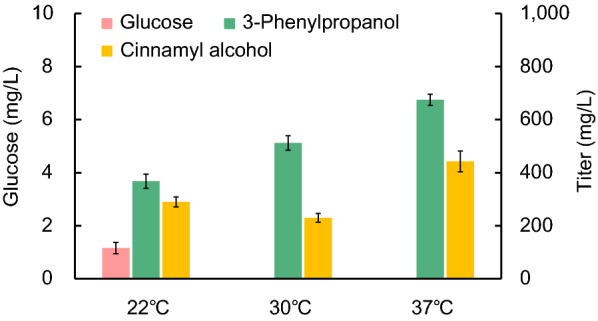
Improvement of 3-phenylpropanol production by optimizing fermentation temperature. Strain BTR31 was cultivated at 22 °C, 30 °C and 37 °C, respectively, with supplementation of 10 g/L glucose and 0.01 mM IPTG. The fermentation broth were analyzed at 48 h

Temperature is a critical parameter for cell growth and end product biosynthesis in fermentation process, and we firstly tested its effect on production of 3-phenylpropanol. We carried the fermentation at the temperature of 22 °C, 30 °C and 37 °C, respectively. Compared to fermentation at 30 °C, glucose was not exhausted and the titer of 3-phenylpropanol was dramatically decreased, while cinnamyl alcohol was greatly accumulated, indicating that lower the fermentation temperature to 22 °C resulted in a poor performance of *E. coli* strains and hampered 3-phenylpropanol biosynthesis. Contrarily, the fermentation at 37 °C which is physiological temperature of *E. coli* improved 3-phenylpropanol production with a titer of 674.76 mg/L (Fig. 6), 31.77% increase compared to that at 30 °C, although along with the increase of cinnamyl alcohol accumulation. The results indicated that 3-phenylpropanol fermentation was more suitable to be conducted under the physiological temperature condition.

**Fig. 7:**
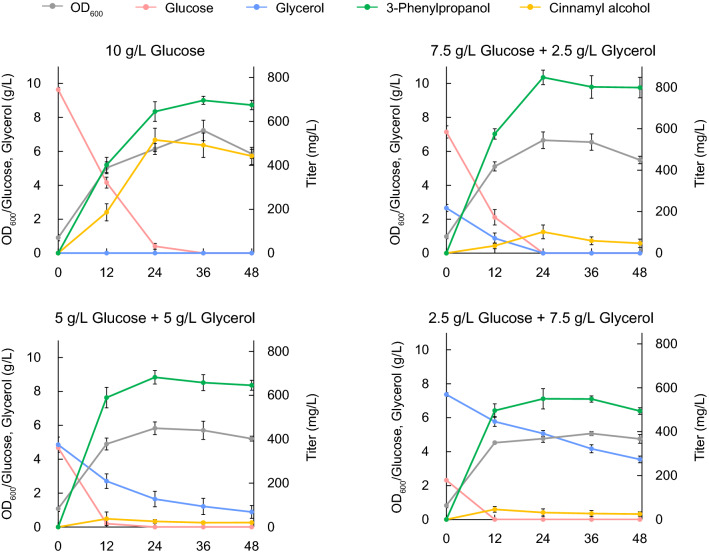
Improvement of 3-phenylpropanol titer by co-utilization of glucose and glycerol. Strain BTR31 was cultivated with glycerol-glucose mixture at different ratios under 37 °C. 0.01 mM IPTG was supplemented. The fermentation broth were analyzed until 48 h

Glycerol is a byproduct of biodiesel production and should therefore be a potential attractive carbon source for the production of valuable chemicals [59]. Then, we tested the effects of taking glycerol with glucose as mixture carbon source on the fermentation of 3-phenylpropanol. As shown in Fig. 7, adding glycerol in mixed medium greatly reduced the accumulation of cinnamyl alcohol, compared to using glucose as the sole carbon source. However, higher ratios of glycerol/glucose (over at 1:1, w/w) had the tendency to reduce the production of 3-phenylpropanol and the utilization of glycerol. When the ratio of glycerol/glucose was at 3:1, 3-phenylpropanol was 549.75 mg/L, 35.17% decrease than that at 1:1. It indicated that glycerol was not directly suitable as a major carbon source because of the inherent carbon mechanism in *E. coli* [60]. Consequently, the combinatorial profit of using glycerol and glucose mixture as co-substrate was achieved with the ratio of 1:3 and the highest amount of 3-phenylpropanol was achieved at a level of 847.79 mg/L, which was 21.82% higher than when glucose was used as the sole carbon source, meanwhile glycerol was completely consumed and the accumulation of unwanted byproduct cinnamyl alcohol was fairly low, representing a high efficiency of fermentation process.

## Conclusions

In present study, a novel 3-phenypropanol biosynthetic pathway was designed by computer-aided retrobiosynthesis analysis, the highest titer for microbial production of 3-phenylpropanol was reported. By dividing the full de novo pathway at l-phenylalanine node, we employed systems metabolic engineering strategies to reconstruct microbial cell factory. We refactored the heterologous downstream pathway comprising AtPAL2, CaER, SruCAR and EcPPTase by changing the different enzymes from microbes and plants, and enhanced the upstream l-phenylalanine pathway by combinatorial chromosome engineering with multi-gene disruption. Tuning the compatibility between chassis strains and the designed downstream pathway, and balancing the expression pattern of pathway genes, resulted in an engineered *E. coli* strain, which produced 847.97 mg/L of 3-phenylpropanol under optimal fermentation conditions. Compared to previous report in which 3-phenypropanol was produced by *S. cerevisiae* [24], a three-fold increase of titer was achieved in our study. This work show the potential for novel metabolic pathway design of bio-based products via retrobiosynthesis approach, which could eliminate the gaps in pathway engineering caused by unidentified enzymes.

## Materials and methods

### In silico biodesign of artificial 3-phenylpropanol pathway

We depicted four 3-phenylpropanol biosynthetic pathways by RetroPath 2.0 [33]. Three input files, Source, Sink and Rules, were required for computation and output of pathways. The Source described the target compound 3-phenylpropanol. The Sink we used was derived from a Sink file that contains compounds from *E. coli* core metabolism ([61], https://github.com/brsynth/RetroPathRL). We added two compounds, phenylpyruvate and l-phenylalanine, to depict putative pathways extending from *E. coli* native l-phenylalanine metabolism. For generation of pathway I and II, we applied the Rules available at https://retrorules.org/dl, release rr02 [34], which contained a complete set of reactions extracted from public databases. For previously reported pathway III and IV, the Rules was encoded based on the involved reactions and generated by RetroRules online rule builder ([34], https://retrorules.org/diy). The parameters were predefined as follows for highly specific predictions: minimum rule diameter was ten, maximum pathway length was six, and number of structures to keep for next iteration was 20.

### Chemicals and reagents

3-Phenylpropanol (99% purity) was purchased from Heowns (China), cinnamic acid (99.5% purity) was purchased from Haoshengjie Biotech (China), 3-phenylpropionic acid (99.88% purity) and 3-phenylpropyl aldehyde (97% purity) were purchased from Bidepharm (China), and cinnamyl alcohol (98% purity) and l-phenylalanine (98% purity) were purchased from Dingguo Changsheng Biotech (China). Acetonitrile, trifluoroacetic acid and ethyl acetate (HPLC grade) were purchased from Tianjin Jiangtian Chemical Technology (China). Phanta Max Super-Fidelity DNA Polymerase and *Taq* Polymerase using in polymerase chain reaction (PCR), and ClonExpress Ultra One Step Cloning Kit applied for plasmid construction were bought from Vazyme (Nanjing, China). Restriction endonucleases and T4 DNA ligase for plasmid construction were purchased from Thermo Scientific (Beijing, China). PCR primers were synthesized by GENEWIZ (Suzhou, China) and listed in Additional file 1: Table S1.

### Plasmids and strains construction

The plasmids and strains used in this study were listed in Table 1.

**Table 1:** Bacterial strains and plasmids used in this study

Name	Characteristics	Sources
Strains		
*E. coli* BL21(DE3)	F^−^ *ompT hsdSB*(*rB*^*−*^* mB*^*−*^) *gal dcm*(DE3)	Invitrogen
*E. coli* BW25113	*lacI*^q^*rrnB*_T14_Δ*lacZ*_WJ16_ *hsdR514* Δ*araBAD*_AH33_ Δ*rhaBAD*_LD78_	NBRP-*E. coli* at NIG
BWH18	BW25113, Δ*feaB*::P_trc_*-aroG*^*fbr*^*-pheA*^*fbr*^, Δ*tyrA*	[51]
BTR01	BL21(DE3) with pQFA01 and pQFA02	This study
BTR02	BL21(DE3) with pQFA01 and pQFA03	This study
BTR03	BL21(DE3) with pQFA01 and pQFA04	This study
BTR04	BL21(DE3) with pQFA01 and pQFA05	This study
BTR05	BL21(DE3) with pQFA01 and pQFA06	This study
BTR06	BL21(DE3) with pQFA07 and pQFA02	This study
BTR07	BL21(DE3) with pQFA08 and pQFA02	This study
BTR08	BTR01 with pQFA09	This study
BTR09	BTR01 with pQFA10	This study
BTR10	BTR01 with pQFA11	This study
BTR11	BTR01 with pQFA12	This study
BTR12	BTR01 with pQFA13	This study
BTR13	BWH18 with T7 RNA polymerase gene in the chromosome	This study
BTR14	BTR13, *∆ptsG*	This study
BTR15	BTR14, *∆pykA*	This study
BTR16	BTR15, *∆pykF*	This study
BTR17	BTR14, *∆tyrR*	This study
BTR18	BTR15, *∆tyrR*	This study
BTR19	BTR16, *∆tyrR*	This study
BTR20	BTR13 with pQFA01, pQFA02 and pQFA10	This study
BTR21	BTR14 with pQFA01, pQFA02 and pQFA10	This study
BTR22	BTR15 with pQFA01, pQFA02 and pQFA10	This study
BTR23	BTR16 with pQFA01, pQFA02 and pQFA10	This study
BTR24	BTR17 with pQFA01, pQFA02 and pQFA10	This study
BTR25	BTR18 with pQFA01, pQFA02 and pQFA10	This study
BTR26	BTR19 with pQFA01, pQFA02 and pQFA10	This study
BTR27	BTR16 with P_T7_-*EcPPTase* fragment integrated into the locus between *entD* and *insL1*	This study
BEL09	BTR27, pCDF-CaER, pQFA02, pQFA10	This study
BTR28	BTR27 with pQFA10 and pQFA14	This study
BTR29	BTR27 with pQFA15 and pQFA02	This study
BTR30	BTR27 with pQFA15 and pQFA16	This study
BTR31	BTR27 with pQFA17 and pQFA18	This study
BTR32	BTR27 with pQFA19 and pQFA20	This study
BTR33	BTR27 with pQFA21 and pQFA22	This study
BTR34	BTR27 with pQFA21 and pQFA16	This study
Plasmids		
pCDFDuet-1	CDF ori with P_T7_; Str^R^	Novagen
pETDuet-1	ColE1 ori with P_T7_; Amp^R^	Novagen
pRSFDuet-1	RSF ori with P_T7_; Kan^R^	Novagen
pREDCas9	pSC101^ts^, pLac-λ-Red, Cas9, P_araBAD_ -gRNA-*bla* Spc^R^	[62]
pGRB	pUC18 for gRNA construction	[62]
pQFA01	pCDFDuet-1 harboring genes encoding CaER and EcPPTase	This study
pQFA02	pETDuet-1 harboring the gene encoding SruCAR	This study
pQFA03	pETDuet-1 harboring the gene encoding MsCAR	This study
pQFA04	pETDuet-1 harboring the gene encoding SroCAR	This study
pQFA05	pETDuet-1 harboring the gene encoding TtCAR	This study
pQFA06	pETDuet-1 harboring the gene encoding NcCAR	This study
pQFA07	pCDFDuet-1 harboring genes encoding CaER and BsPPTase	This study
pQFA08	pCDFDuet-1 harboring genes encoding CaER and NiPPTase	This study
pQFA09	pRSFDuet-1 harboring the gene encoding TpPAL1	This study
pQFA10	pRSFDuet-1 harboring the gene encoding AtPAL2	This study
pQFA11	pRSFDuet-1 harboring the gene encoding ZmPAL2	This study
pQFA12	pRSFDuet-1 harboring the gene encoding RgTAL	This study
pQFA13	pRSFDuet-1 harboring the gene encoding SriPAL	This study
pQFA14	pETDuet-1 harboring genes encoding SruCAR and CaER	This study
pQFA15	pRSFDuet-1 harboring genes encoding AtPAL2 and CaER	This study
pQFA16	pCDFDuet-1 harboring the gene encoding SruCAR	This study
pQFA17	pETDuet-1 harboring the gene encoding AtPAL2	This study
pQFA18	pRSFDuet-1 harboring genes encoding SruCAR and CaER	This study
pQFA19	pETDuet-1 harboring genes encoding SruCAR and AtPAL2	This study
pQFA20	pRSFDuet-1 harboring the gene encoding CaER	This study
pQFA21	pETDuet-1 harboring genes encoding AtPAL2 and CaER	This study
pQFA22	pRSFDuet-1 harboring the gene encoding SruCAR	This study

For reconstruction of 3-phenylpropanol downstream pathway, codon-optimized genes encoding candidates CARs, PPTases and PALs were fully synthesized by Genscript (Nanjing, China). The nucleotide sequences of codon optimized genes were listed in Additional file 1: Table S2. The gene encoding CaER was cloned into pCDFDuet-1 at sites of restriction endonucleases *Nco*I and *Bam*HI, resulting an intermediate plasmid pCDF-CaER. The gene encoding EcPPTase was cloned into pCDFDuet-1 using restriction endonucleases *Nde*I and *Bgl*II to generate plasmid pQFA01. Genes encoding SruCAR, MsCAR, SroCAR, TtCAR and NcCAR were cloned into pETDuet-1 using *Nco*I and *Bam*HI to generate plasmids pQFA02, pQFA03, pQFA04, pQFA05, and pQFA06, respectively. Similarly, plasmids pQFA07 and pQFA08 were obtained by cloning genes encoding candidate BsPPTases and NiPPTase into pCDF-CaER at the sites *Nde*I and *Bgl*II, respectively. And plasmids pQFA09, pQFA10 and pQFA11 were obtained by cloning genes encoding TpPAL1, AtPAL2 and ZmPAL2 into plasmid pRSFDuet-1 at the sites *Nco*I and *Bam*HI, respectively. Plasmid pQFA12 were obtained by cloning genes encoding RgTAL into plasmid pRSFDuet-1 at the sites *EcoR*I and *Hin*dIII. Plasmid pQFA13 were obtained by cloning genes encoding SriPAL into plasmid pRSFDuet-1 at the sites *Nde*I and *Bgl*II. *E. coli* BL21 (DE3) was transformed with pQFA01 in combination with pQFA02, pQFA03, pQFA04, pQFA05, and pQFA06 to generated strains BTR01, BTR02, BTR03, BTR04, and BTR05, respectively. Strains BTR06-BTR12 were constructed by co-transforming various combinations of expression plasmids as indicated in Table 1.

For combinatorial chromosome engineering of l-phenylalanine producing strains, genes integration and deletion on *E. coli* BWH18 chromosome [51] were realized by CRISPR-Cas9 meditated genome editing method [62]. In Additional file 1: Table S1, we listed the guide RNA sequences and the primers for construction of corresponding plasmid pGRB and donor DNA. We integrated T7 RNA polymerase gene into the locus between *ybhC* and *ybhB* on BWH18 chromosome to obtain strain BTR13. We sequentially deleted genes *ptsG*, *pykA*, *pykF* in BTR13 chromosome to generate strains BTR14, BTR15 and BTR16, respectively. The gene *tyrR* was disrupted in the chromosome of strains BTR14, BTR15 and BTR16 to generate strains BTR17, BTR18 and BTR19, respectively. For de novo production of 3-phenylpropanol, three plasmids pQFA01, pQFA02 and pQFA10, were cotransformed into strains BTR13-BTR19 to construct BTR20-BTR26, respectively.

For metabolic strength balancing of downstream 3-phenylpropanol pathway, the gene encoding EcPPTase under T_7_ promoter was cloned from pQFA01 and integrated into the locus between *entD* and *insL1* on BTR16 chromosome to obtain strain BTR27, according to CRISPR-Cas9 meditated genome editing method [62]. The pathway genes encoding AtPAL2, CaER and SruCAR were modulated in plasmids pRSFDuet-1, pETDuet-1 or pCDFDuet-1. We used a two-fragment assembling method for construction of plasmids pQFA14-pQFA22 according to the guideline of ClonExpress Ultra One Step Cloning Kit (Vazyme, Nanjing, China), and the primers were listed in Additional file 1: Table S1. All expression vectors harboring two genes were constructed in bicistronic pattern. Strains BTR28-BTR34 were constructed by cotransformation of two expression vectors harboring all three pathway genes (Fig. 5).

### Cultivation media and conditions

For strain cultivation and seed preparation, Luria broth (LB) medium containing 10 g/L tryptone, 5 g/L yeast extract, and 10 g/L NaCl was used. For fermentations, M9 medium containing 17.1 g/L Na_2_HPO_4_·12H_2_O, 3.0 g/L KH_2_PO_4_, 0.5 g/L NaCl, 1.0 g/L NH_4_Cl, 5 mM MgSO_4_, 0.1 mM CaCl_2_ and 2 mg/L vitamin B1 (pH 7.2) was used. 1 g/L and 5 g/L yeast extract were added in M9 medium when *E. coli* BL21(DE3) and *E. coli* BW25113 derived strains were employed as the host, respectively. 200 mg/L cinnamic acid was added for screening CARs and PPTases. 500 mg/L l-phenylalanine was added for screening PALs. Antibiotics were added to the medium as following concentrations when needed: 100 μg/mL ampicillin, 30 μg/mL streptomycin and 30 μg/mL kanamycin.

For fermentation experiments, bacterial clones were transferred into 5 mL LB medium and cultivated at 37 °C and 220 rpm. The overnight culture was diluted at 1:100 into 25 mL LB medium of 250 mL shake-flask and cultivated for 6–8 h (37 °C, 220 rpm). Then cells were collected by centrifugation and resuspended into 25 mL of M9Y medium at an initial OD_600_ of 1. The fermentation was performed under 30 °C and 220 rpm with 10 g/L glucose and 0.1 mM IPTG supplemented if not indicated. For optimization of culture conditions, the fermentation was carried out under different temperatures and supplemented with glucose-glycerol mixture at designed ratios. The experiments were carried out in triplicates and the data was shown as means ± S.D.

### Biomass and metabolite analysis

Cell optical density (OD) was observed at 600 nm using a TU-1810 spectrophotometer. The fermentation broth was sampled by directly centrifuged, then the supernatant was filtered and analyzed by a Hitachi Primaide HPLC system (Japan). 3-Phenylpropanol, cinnamic acid, 3-phenylpropionic acid, 3-phenylpropyl aldehyde, cinnamyl alcohol and l-phenylalanine were separated by a Thermo Scientific Hypersil BDS C18 column (150 × 4.6 mm, 5 μm) and measured by a PDA detector at 210 nm with a mobile phase (20% acetonitrile, 80% water, 0.1% trifluoroacetic acid) at 1 mL/min. Glucose and glycerol were measured by a Morphling™ Sugar-H column (300 × 7.8 mm, 5 μm) and a RI detector with a mobile phase (5 mM H_2_SO_4_) at 0.6 mL/min, 65 °C. All of aforementioned compounds were quantified by HPLC analysis using a five-point calibration curve with the R^2^ coefficient higher than 0.99. GC–MS analysis for 3-phenylpropanol identity was conducted by Agilent Technologies gas chromatography-triple quadrupole tendem mass spectrometry 7890B-7000D. The oven temperature was initially held at 50 °C for 4 min. Next the temperature was increased at 5 °C/min to 150 °C and then at 90 °C/min to 250 °C. Temperatures of the injection port and the ionizing source were 250 °C and 280 °C, respectively. The split ratio was 10:1 and 1 µL of sample was injected.

## Supplementary Information

**Additional file 1: Table S1.** The main primers used in this study. **Table S2.** Nucleotide sequences of codon optimized genes used in this study. **Figure S1.** GC–MS analysis for identification of 3-phenylpropanol.

## Data Availability

All data generated or analyzed during this study are included in this article and in the Additional file 1.
