# Supplementary material for: Metabolic engineering of Escherichia coli for de novo production of 3-phenylpropanol via retrobiosynthesis approach
Source: Microb Cell Fact. 2021 Jun 27;20:121. doi: 10.1186/s12934-021-01615-1 (PMC8237410; doi:10.1186/s12934-021-01615-1)
Supplement: Supplementary file 1 — Additional file 1: Table S1. The main primers used in this study. Table S2. Nucleotide sequences of codon optimized genes used in this study. Figure S1. GC–MS analysis for identification of 3-phenylpropanol. [file 12934_2021_1615_MOESM1_ESM.docx]

*Research Article*

Metabolic engineering of *Escherichia coli* for *de novo* production of 3-phenylpropanol via retrobiosynthesis approach

Zhenning Liu^†^, Xue Zhang^†^, Dengwei Lei^†^, Bin Qiao^†^, Guang-Rong Zhao^*,†,‡^

^†^Frontier Science Center for Synthetic Biology and Key Laboratory of Systems Bioengineering (Ministry of Education), School of Chemical Engineering and Technology, Tianjin University, Yaguan Road 135, Jinnan District, Tianjin 300350, China

^‡^Georgia Tech Shenzhen Institute, Tianjin University, Tangxing Road 133, Nanshan District, Shenzhen, 518071, China

^*^Corresponding author: Guang-Rong Zhao (grzhao@tju.edu.cn)

Tel: +86-22-85356580; Fax: +86-22-27403389

**Additional file 1：**

**Table S1:** The main primers used in this study.

**Table S2:** Nucleotide sequences of codon optimized genes used in this study.

**Fig. S1:** GC-MS analysis for identification of 3-phenylpropanol.

**Table S1.** The main primers used in this study.

| Primer | Sequence |
| --- | --- |
| 3-Phenylpropanol pathway reconstruction | |
| CaER F | GATATACCATGGGCATGAACAAATACAAGAAACTGTTCGAACCGA |
| CaER R | GGATCCTTATTAGATGTGGTTCGCAACCTCAAACG |
| pCDF F | GCGAACCACATCTAATAAGGATCCGAATTCGAGCTCGGC |
| pCDF R | GTTCATGCCCATGGTATATCTCCTTATTAAAGTTAAACAAAATTATTTC |
| Chromosome engineering | |
| gRNA-T7RP | CTAACTTGAGCGAAACGGGA |
| T7RP-P1 | ATCAAGGGAAAGCCCAATCTTCACATC |
| T7RP-P2 | ATTGCCGCATCGTCATGTCTTT |
| gRNA- ptsG | GCGCTGACCTCGTTCCTGAC |
| ptsG-P1 | CTGACTCACCTTACCTTGCGCC |
| ptsG-P2 | GGTTACGGATGTACTCCATAATTGAGAGTGCTCCTGAGTATGGGT |
| ptsG-P3 | TCAATTATGGAGTACATCCGTAACCACTAATCCGTAAGAC |
| ptsG-P4 | CGCAATTACCGACAACTGGCAG |
| gRNA-pykA | CGTTTGCAGCCAGGATGCAA |
| pykA-P1 | ACGGAGACGTGGTGGTGCTGA |
| pykA-P2 | TACGCATGTAATACTCCGTTGACTGAAACAACCAGGT |
| pykA-P3 | TCAACGGAGTATTACATGCGTATTTTAACGGTAGAGTAAGTACGTTGCCGGA |
| pykA-P4 | TCATGGCCCAGAGCACAGCGA |
| gRNA-pykF | CTGATCTTTGGTTGCGAACA |
| pykF-P1 | ACTTATCCTCACACTGACAACTTCGGCA |
| pykF-P2 | TGAACAGATGCCATGACAGTCTTAGTCTTTAAGTTGAGAAGGATGGGAG |
| pykF-P3 | ACTGTCATGGCATCTGTTCACGTCCTGTAATATTGCTTTTGT |
| pykF-P4 | AGCTGCGTCATCTTTAGCAGCCT |
| gRNA-tyrR | CCGCAACTTATTGGCAATCG |
| tyrR-P1 | ATCGCATCGCCACGCTGTATG |
| tyrR-P2 | CGTTCTTCTTCTGCATGGGAACCTTCACCTGAAAAAAGAACA |
| tyrR-P3 | TTCCCATGCAGAAGAAGAACGAAGAGTAAGCGCGAA |
| tyrR-P4 | GATCTGTCTGACGTCACCCTCGGT |
| Expression strength balancing | |
| pQFA14-15-P1 | GGAATTCCATATGAACAAATACAAGAAACTGTTCGAACCGAT |
| pQFA14-15-P2 | CGGGGTACCTTATTAGATGTGGTTCGCAACCTCAAACG |
| pQFA16-18-22-P1 | TACCATGGGCATGACCGAGAGCCA |
| pQFA16-22-P2 | GAGCTCGAATTCGGATCCTTATTACAGCA |
| pQFA16-22-P3 | TGCTGTAATAAGGATCCGAATTCGAGCTCGG |
| pQFA16-18-22-P4 | TCTCGGTCATGCCCATGGTATATCTCCTTATTAAAGTTAAAC |
| pQFA17-21-P1 | ACCATGGGCATGGACCAGATCG |
| pQFA17-P2 | CGAATTCGGATCCTTATTAGCAAATCGGGA |
| pQFA17-P3 | GCTAATAAGGATCCGAATTCGAGCTCGG |
| pQFA17-21-P4 | ATCTGGTCCATGCCCATGGTATATCTCCTTCTTAAAG |
| pQFA18-21-P2 | CGAGGGTACCTTATTAGATGTGGTTCGCA |
| pQFA18-21-P3 | CATCTAATAAGGTACCCTCGAGTCTGGTAAAGAAAC |
| pQFA19-P1 | ACATATGGACCAGATCGAAGCGATGCT |
| pQFA19-P2 | ACTCGAGGGTACCTTATTAGCAAATCGGGATCGGC |
| pQFA19-P3 | GCTAATAAGGTACCCTCGAGTCTGGTAAAG |
| pQFA19-P4 | CTTCGATCTGGTCCATATGTATATCTCCTTCTTATACTTAACTAATATACTAAGATGG |
| pQFA20-P1 | ACCATGGGCATGAACAAATACAAGAAACT |
| pQFA20-P2 | CGGATCCTTATTAGATGTGGTTCGCAAC |
| pQFA20-P3 | CATCTAATAAGGATCCGAATTCGAGCTCGG |
| pQFA20-P4 | GTATTTGTTCATGCCCATGGTATATCTCCTTATTAAAGTTAAAC |

**Table S2.** Nucleotide sequences of codon optimized genes used in this study.

| *CaER* | ATGAACAAATACAAGAAACTGTTCGAACCGATCAAGATTGGTAAATGCGAGATCAAGAACCGTTTTGCGCTGGCGCCGATGGGTCCGCTGGGTCTGGCGGACAGCGAAGGTGGCTTCAACCAGCGTGGTATTGATTACTATACCGAGCGTGCGAAAGGTGGCACCGGTCTGATCATTACCGGCGTGACCTTCGTTGACAACGAAGTGGAGGAACACGGTATGCCGAACTGCCCGTGCCCGACCCACAACCCGGTGCAGTTTGTTCGTACCGGCCGTGAAATGACCGAGCGTATCCACGCGTATAACAGCAAGGTGTTCCTGCAAATGAGCGGTGGCTTTGGTCGTGTTACCATCCCGACCAACCTGGGTGAGTTTCCGCCGGTTGCGCCGAGCCCGATTCAACACCGTTGGCTGGACAAAACCTGCCGTGAACTGACCGTGGATGAGATCAAGAGCATTGTTAAGAAATTCGGTGAAGGCGCGTTTAACGCGAAACGTGCGGGTTTCGACGGCGTGCAGATCCACGCGGTTCACGAGGGTTACCTGATCGACCAATTCGCGATTAGCCTGTTTAACCACCGTACCGATGAATATGGTGGCAGCCTGGAGAACCGTCTGCGTTTTGCGCGTGAAATCGTGGAGGAAATTAAGAACCGTTGCGGCGAGGATTTCCCGGTTACCCTGCGTTACAGCCCGAAGAGCTTCATTAAGGACCTGCGTGATGGTGCGCTGCCGGGCGAGGAGTTCGTGGAAAAAGGTCGTGACCTGGATGAAGGCGTGGAGGCGGCGAAGCTGCTGGTTAGCTACGGTTATGACGCGCTGGACACCGATGTTGGCAGCTACGATTCCTGGTGGTGGAGCCACCCGCCGATGTATCAGGAGAAAGGTCTGTACCGTAAGTATGCGAAACTGATGAAGGACACCGTTGATGTGCCGGTTATCTGCGCGGGTCGTATGGACGATCCGGACATGGCGCTGGAAGCGGTGGAGAACGGCACCTGCGATGTTATTAGCCTGGGCCGTCCGCTGCTGGCGGACCCGGATTACGTGAACAAGCTGCGTAGCAACAAGTGCAAAAGCATCCGTCCGTGCATTAGCTGCCAGGAAGGTTGCATGGGCCGTGTGCAACACTATAGCATGCTGAACTGCGCGGTTAACCCGCAAGCGTGCAAAGAGCGTGCGAACAGCCTGACCCCGATCATTAAGAGCAAGAAAGTGCTGATCGTTGGTGGCGGTGTGGCGGGTTGCGAAGCGGCGCGTGTTCTGGCGCTGCGTGGTCACGAACCGGTTCTGTACGAGAAAAGCAACCGTCTGGGCGGTAACCTGATCCCGGGCGGTGCGCCGAGCTTCAAAGAGGACGATATTGCGCTGGCGGACTGGTATACCAACACCCTGAAGGAACTGAACGTTGAGGTGAACCTGAACAGCGAAGTGACCAAAGAGCAGATCCTGAACAGCAAGTTTGATACCGTGATTGTTGCGACCGGTAGCACCCCGAAAGTTTTCCCGCTGGGCGACGATGAAAAGGTGTTTACCGCGGCGGAAGTGCTGCTGGGTCAAAAAGACCCGGGTGAAACCACCGTGGTTGTGGGCGGTGGCCTGGTGGGTTGCGAGCTGGCGCTGGATCTGGCGAAGAAAGGCAAGAAAGTGACCATCGTTGAAGCGCTGAACAAGATTCTGGCGCTGAACGGTCCGCTGTGCAGCGCGAACAGCGAGATGCTGCAAAAACTGATCCCGTTCAACGGCATTGACGTGAAGGCGAACAGCAAGGTTAAAGGTTACAAAAACGGCCTGCTGAAGATGGAGACCGAAAACGGTATCGAGGAACTGCCGTGCGATAGCGTGATTCTGAGCGTTGGCTACAAAGAGGAAAACAGCCTGTATAAGGAGCTGGAGTTCGAGATCCCGGAAATTTACCTGCTGGGTGACGCGCGTAAAGTGAGCAACATCATGTATGGCATTTGGGATGCGTTTGAGGTTGCGAACCACATCTAA |
| --- | --- |
| *EcPPTase* | ATGGTGGACATGAAAACCACCCACACCAGCCTGCCGTTTGCGGGTCACACCCTGCACTTCGTTGAGTTTGACCCGGCGAACTTTTGCGAACAGGATCTGCTGTGGCTGCCGCACTATGCGCAGCTGCAACATGCGGGTCGTAAGCGTAAAACCGAGCACCTGGCGGGTCGTATTGCGGCGGTGTATGCGCTGCGTGAATACGGTTATAAATGCGTTCCGGCGATTGGTGAACTGCGTCAACCGGTGTGGCCGGCGGAAGTTTACGGTAGCATCAGCCATTGCGGCACCACCGCGCTGGCGGTGGTTAGCCGTCAGCCGATCGGCATTGACATCGAGGAAATCTTCAGCGTGCAAACCGCGCGTGAGCTGACCGACAACATCATTACCCCGGCGGAGCACGAACGTCTGGCGGATTGCGGTCTGGCGTTTAGCCTGGCGCTGACCCTGGCGTTCAGCGCGAAGGAGAGCGCGTTTAAAGCGAGCGAAATTCAGACCGACGCGGGCTTCCTGGATTATCAAATCATTAGCTGGAACAAGCAGCAAGTTATCATTCACCGTGAGAACGAAATGTTTGCGGTGCACTGGCAGATCAAGGAAAAAATTGTTATCACCCTGTGCCAACACGATTAA |
| *SruCAR* | ATGACCGAGAGCCAGAGCTACGAAACCCGTCAAGCGCGTCCGGCGGGTCAGAGCCTGGCGGAGCGTGTGGCGCGTCTGGTTGCGATTGACCCGCAGGCGGCGGCGGCGGTGCCGGATAAAGCGGTTGCGGAGCGTGCGACCCAGCAAGGTCTGCGTCTGGCGCAACGTATCGAAGCGTTCCTGAGCGGTTATGGCGACCGTCCGGCGCTGGCGCAGCGTGCGTTTGAGATCACCAAAGATCCGATCACCGGTCGTGCGGTGGCGACCCTGCTGCCGAAGTTCGAAACCGTTAGCTATCGTGAGCTGCTGGAACGTAGCCACGCGATTGCGAGCGAGCTGGCGAACCATGCGGAAGCGCCGGTGAAAGCGGGCGAGTTCATCGCGACCATTGGTTTTACCAGCACCGACTACACCAGCCTGGATATTGCGGGCGTGCTGCTGGGTCTGACCAGCGTTCCGCTGCAAACCGGTGCGACCACCGACACCCTGAAAGCGATTGCGGAGGAAACCGCGCCGGCGGTGTTTGGTGCGAGCGTTGAACACCTGGACAACGCGGTTACCACCGCGCTGGCGACCCCGAGCGTGCGTCGTCTGCTGGTTTTTGATTATCGTCAGGGTGTGGACGAGGATCGTGAAGCGGTTGAAGCGGCGCGTAGCCGTCTGGCGGAGGCGGGTAGCGCGGTGCTGGTTGACACCCTGGATGAAGTGATCGCGCGTGGCCGTGCGCTGCCGCGTGTTGCGCTGCCGCCGGCGACCGACGCGGGTGACGATAGCCTGAGCCTGCTGATTTACACCAGCGGTAGCACCGGCACCCCGAAGGGTGCGATGTATCCGGAGCGTAACGTGGCGCAGTTCTGGGGTGGCATCTGGCACAACGCGTTTGACGATGGTGATAGCGCGCCGGACGTGCCGGATATCATGGTTAACTTCATGCCGCTGAGCCACGTTGCGGGTCGTATTGGCCTGATGGGCACCCTGAGCAGCGGTGGCACCACCTACTTTATCGCGAAAAGCGACCTGAGCACCTTCTTTGAGGATTATAGCCTGGCGCGTCCGACCAAGCTGTTCTTTGTGCCGCGTATCTGCGAGATGATTTACCAGCACTATCAAAGCGAACTGGACCGTATTGGTGCGGCGGATGGTAGCCCGCAAGCGGAAGCGATTAAAACCGAACTGCGTGAGAAGCTGCTGGGTGGCCGTGTTCTGACCGCGGGTAGCGGCAGCGCGCCGATGAGCCCGGAGCTGACCGCGTTCATTGAAAGCGTTCTGCAGGTGCACCTGGTTGACGGTTATGGCAGCACCGAAGCGGGTCCGGTGTGGCGTGATCGTAAGCTGGTGAAACCGCCGGTTACCGAGCACAAACTGATCGACGTTCCGGAACTGGGCTACTTTAGCACCGATAGCCCGTATCCGCGTGGTGAGCTGGCGATCAAAACCCAAACCATTCTGCCGGGTTACTATAAGCGTCCGGAAACCACCGCGGAAGTGTTCGACGAAGATGGCTTTTACCTGACCGGTGACGTGGTTGCGGAAGTTGCGCCGGAGGAGTTCGTGTATGTTGATCGTCGTAAAAACGTGCTGAAGCTGAGCCAGGGCGAGTTTGTTGCGCTGAGCAAGCTGGAAGCGGCGTATGGCACCAGCCCGCTGGTGCGTCAAATTAGCGTTTACGGTAGCAGCCAGCGTAGCTATCTGCTGGCGGTGGTTGTGCCGACCCCGGAGGCGCTGGCGAAATACGGTGATGGTGAAGCGGTGAAGAGCGCGCTGGGTGATAGCCTGCAAAAAATCGCGCGTGAGGAAGGTCTGCAGAGCTATGAGGTTCCGCGTGACTTCATCATTGAAACCGACCCGTTTACCATCGAAAACGGCATTCTGAGCGACGCGGGCAAGACCCTGCGTCCGAAGGTGAAAGCGCGTTACGGCGAACGTCTGGAGGCGCTGTATGCGCAGCTGGCGGAAACCCAAGCGGGTGAACTGCGTAGCATTCGTGTGGGTGCGGGTGAGCGTCCGGTGATTGAAACCGTTCAACGTGCTGCGGCGGCGCTGCTGGGTGCGAGCGCGGCGGAAGTGGACCCGGAAGCGCACTTCAGCGACCTGGGTGGCGATAGCCTGAGCGCGCTGACCTACAGCAACTTCCTGCACGAAATCTTCCAGGTGGAAGTTCCGGTGAGCGTTATTGTGAGCGCGGCGAACAACCTGCGTAGCGTGGCGGCGCACATCGAGAAAGAACGTAGCAGCGGTAGCGACCGTCCGACCTTTGCGAGCGTTCACGGTGCGGGTGCGACCACCATTCGTGCGAGCGACCTGAAGCTGGAGAAATTTCTGGATGCGCAAACCCTGGCTGCGGCGCCGAGCCTGCCGCGTCCGGCGAGCGAAGTGCGTACCGTTCTGCTGACCGGCAGCAACGGTTGGCTGGGCCGTTTCCTGGCGCTGGCGTGGCTGGAGCGTCTGGTGCCGCAGGGTGGCAAGGTTGTGGTTATTGTTCGTGGCAAGGACGATAAAGCGGCGAAGGCGCGTCTGGACAGCGTGTTCGAAAGCGGTGATCCGGCGCTGCTGGCGCACTATGAGGACCTGGCGGATAAAGGCCTGGAAGTTCTGGCGGGTGACTTTAGCGATGCGGATCTGGGCCTGCGTAAGGCGGACTGGGATCGTCTGGCGGACGAGGTGGATCTGATCGTTCACAGCGGTGCGCTGGTGAACCACGTTCTGCCGTACAGCCAGCTGTTCGGCCCGAACGTGGTTGGCACCGCGGAAGTGGCGAAACTGGCGCTGACCAAACGTCTGAAGCCGGTTACCTATCTGAGCACCGTTGCGGTGGCGGTTGGCGTGGAACCGAGCGCGTTTGAGGAAGACGGTGATATCCGTGACGTGAGCGCGGTTCGTAGCATTGATGAGGGCTACGCGAACGGTTATGGCAACAGCAAGTGGGCGGGTGAAGTGCTGCTGCGTGAAGCGTACGAGCATGCGGGCCTGCCGGTTCGTGTGTTCCGTAGCGACATGATCCTGGCGCACCGTAAATATACCGGCCAACTGAACGTTCCGGATCAGTTCACCCGTCTGATCCTGAGCCTGCTGGCGACCGGTATTGCGCCGAAGAGCTTTTATCAGCTGGATGCGACCGGTGGCCGTCAGCGTGCGCACTATGACGGTATCCCGGTGGATTTCACCGCGGAAGCGATTACCACCCTGGGTCTGGCGGGCAGCGATGGTTACCACAGCTTCGATGTGTTTAACCCGCACCACGATGGTGTTGGCCTGGACGAGTTTGTGGATTGGCTGGTTGAAGCGGGCCACCCGATCAGCCGTGTGGACGATTATGCGGAGTGGCTGAGCCGTTTCGAAACCAGCCTGCGTGGTCTGCCGGAGGCGCAGCGTCAACACAGCGTTCTGCCGCTGCTGCACGCGTTTGCGCAGCCGGCGCCGGCGATTGATGGCAGCCCGTTCCAGACCAAAAACTTTCAGAGCAGCGTGCAAGAGGCGAAGGTTGGTGCGGAACACGACATTCCGCACCTGGATAAAGCGCTGATCGTTAAGTACGCGGAAGATATTAAACAGCTGGGTCTGCTGTAA |
| *MsCAR* | ATGCACCAGCTGACCGTTACCGGCATGAACATCTGCGAGGTGCAACGTCTGTTCCCGCGTATGACCAGCGACGTTCACGATGCGACCGACGGTGTGACCGAAACCGCGCTGGACGATGAACAGAGCACCCGTCGTATCGCGGAGCTGTACGCGACCGATCCGGAGTTCGCGGCGGCGGCGCCGCTGCCGGCGGTGGTTGACGCGGCGCACAAGCCGGGCCTGCGTCTGGCGGAAATTCTGCAAACCCTGTTTACCGGTTACGGCGATCGTCCGGCGCTGGGTTATCGTGCGCGTGAGCTGGCGACCGATGAAGGTGGCCGTACCGTTACCCGTCTGCTGCCGCGTTTCGATACCCTGACCTATGCGCAAGTGTGGAGCCGTGTTCAAGCGGTGGCTGCGGCGCTGCGTCACAACTTTGCGCAACCGATCTACCCGGGTGATGCGGTTGCGACCATTGGTTTTGCGAGCCCGGATTACCTGACCCTGGACCTGGTGTGCGCGTATCTGGGTCTGGTTAGCGTGCCGCTGCAACATAACGCGCCGGTTAGCCGTCTGGCGCCGATCCTGGCGGAAGTGGAACCGCGTATTCTGACCGTGAGCGCGGAGTACCTGGATCTGGCGGTTGAAAGCGTGCGTGACGTTAACAGCGTGAGCCAGCTGGTTGTGTTCGATCACCACCCGGAAGTGGATGATCACCGTGATGCGCTGGCGCGTGCGCGTGAACAACTGGCGGGCAAGGGCATCGCGGTGACCACCCTGGATGCGATTGCGGATGAGGGTGCGGGCCTGCCGGCGGAACCGATCTACACCGCGGACCACGATCAGCGTCTGGCGATGATTCTGTATACCAGCGGTAGCACCGGTGCGCCGAAAGGTGCGATGTACACCGAGGCGATGGTTGCGCGTCTGTGGACCATGAGCTTCATCACCGGTGACCCGACCCCGGTTATTAACGTGAACTTTATGCCGCTGAACCACCTGGGTGGCCGTATCCCGATTAGCACCGCGGTTCAAAACGGTGGCACCAGCTATTTCGTGCCGGAGAGCGATATGAGCACCCTGTTTGAAGACCTGGCGCTGGTGCGTCCGACCGAACTGGGTCTGGTTCCGCGTGTGGCGGACATGCTGTACCAGCACCACCTGGCGACCGTTGATCGTCTGGTGACCCAGGGTGCGGATGAGCTGACCGCGGAAAAGCAGGCGGGTGCGGAGCTGCGTGAACAAGTTCTGGGTGGCCGTGTGATCACCGGCTTCGTTAGCACCGCGCCGCTGGCGGCGGAGATGCGTGCGTTTCTGGATATCACCCTGGGTGCGCACATTGTTGACGGTTATGGCCTGACCGAAACCGGTGCGGTGACCCGTGACGGTGTTATTGTGCGTCCGCCGGTTATCGATTACAAGCTGATTGACGTGCCGGAGCTGGGCTACTTCAGCACCGATAAACCGTATCCGCGTGGTGAACTGCTGGTTCGTAGCCAGACCCTGACCCCGGGTTACTATAAGCGTCCGGAAGTGACCGCGAGCGTGTTTGACCGTGATGGCTACTATCACACCGGTGATGTGATGGCGGAAACCGCGCCGGATCACCTGGTTTATGTGGACCGTCGTAACAACGTTCTGAAACTGGCGCAGGGCGAGTTCGTTGCGGTGGCGAACCTGGAAGCGGTGTTTAGCGGTGCGGCGCTGGTGCGTCAAATCTTCGTTTATGGTAACAGCGAGCGTAGCTTTCTGCTGGCGGTGGTTGTGCCGACCCCGGAGGCGCTGGAACAGTATGACCCGGCGGCGCTGAAAGCGGCGCTGGCGGATAGCCTGCAGCGTACCGCGCGTGACGCGGAGCTGCAAAGCTACGAAGTTCCGGCGGATTTCATCGTGGAAACCGAACCGTTTAGCGCGGCGAACGGCCTGCTGAGCGGTGTGGGCAAGCTGCTGCGTCCGAACCTGAAAGATCGTTACGGTCAGCGTCTGGAGCAAATGTATGCGGACATTGCGGCGACCCAGGCGAACCAACTGCGTGAGCTGCGTCGTGCTGCGGCGACCCAGCCGGTTATCGACACCCTGACCCAAGCTGCGGCGACCATTCTGGGCACCGGCAGCGAAGTGGCGAGCGATGCGCACTTCACCGATCTGGGTGGCGACAGCCTGAGCGCGCTGACCCTGAGCAACCTGCTGAGCGACTTCTTTGGCTTTGAGGTTCCGGTGGGCACCATCGTTAACCCGGCGACCAACCTGGCGCAGCTGGCGCAACACATTGAAGCGCAACGTACCGCGGGCGATCGTCGTCCGAGCTTCACCACCGTGCACGGTGCGGATGCGACCGAGATCCGTGCGAGCGAACTGACCCTGGATAAGTTTATTGATGCGGAAACCCTGCGTGCGGCGCCGGGCCTGCCGAAAGTTACCACCGAACCGCGTACCGTTCTGCTGAGCGGTGCGAACGGTTGGCTGGGTCGTTTCCTGACCCTGCAATGGCTGGAGCGTCTGGCGCCGGTTGGTGGCACCCTGATCACCATTGTGCGTGGTCGTGACGATGCGGCGGCGCGTGCGCGTCTGACCCAAGCGTACGACACCGATCCGGAGCTGAGCCGTCGTTTTGCGGAACTGGCGGACCGTCACCTGCGTGTTGTGGCGGGCGATATCGGTGACCCGAACCTGGGTCTGACCCCGGAGATTTGGCACCGTCTGGCGGCGGAAGTTGATCTGGTTGTGCATCCGGCGGCGCTGGTTAACCATGTGCTGCCGTATCGTCAGCTGTTCGGCCCGAACGTTGTGGGCACCGCGGAAGTGATCAAGCTGGCGCTGACCGAACGTATTAAACCGGTGACCTACCTGAGCACCGTTAGCGTGGCGATGGGCATCCCGGACTTTGAGGAAGACGGTGATATTCGTACCGTTAGCCCGGTGCGTCCGCTGGATGGTGGCTACGCGAACGGTTATGGCAACAGCAAGTGGGCGGGCGAGGTTCTGCTGCGTGAAGCGCATGATCTGTGCGGTCTGCCGGTGGCGACCTTCCGTAGCGACATGATTCTGGCGCACCCGCGTTACCGTGGTCAGGTTAACGTGCCGGACATGTTCACCCGTCTGCTGCTGAGCCTGCTGATCACCGGTGTTGCGCCGCGTAGCTTTTACATTGGTGATGGTGAGCGTCCGCGTGCGCACTATCCGGGTCTGACCGTTGATTTTGTTGCGGAGGCGGTGACCACCCTGGGTGCGCAGCAACGTGAAGGTTACGTTAGCTATGACGTGATGAACCCGCACGACGATGGCATCAGCCTGGATGTTTTCGTGGACTGGCTGATCCGTGCGGGTCACCCGATTGATCGTGTTGACGATTATGACGATTGGGTGCGTCGTTTTGAAACCGCGCTGACCGCGCTGCCGGAAAAACGTCGTGCGCAAACCGTGCTGCCGCTGCTGCATGCGTTCCGTGCGCCGCAAGCGCCGCTGCGTGGCGCGCCGGAGCCGACCGAAGTGTTTCATGCGGCGGTTCGTACCGCGAAAGTGGGTCCGGGTGATATCCCGCACCTGGACGAGGCGCTGATCGATAAATACATTCGTGACCTGCGTGAGTTCGGTCTGATTTAA |
| *SroCAR* | ATGACCCAGAGCCACACCCAGGGTCCGCAAGCGAGCGCGGCGCACAGCCGTCTGGCGCGTCGTGCGGCGGAGCTGCTGGCGACCGACCCGCAGGCTGCGGCGACCCTGCCGGACCCGGAAGTGGTTCGTCAGGCGACCCGTCCGGGCCTGCGTCTGGCGGAACGTGTGGACGCGATCCTGAGCGGTTATGCGGATCGTCCGGCGCTGGGTCAGCGTAGCTTCCAAACCGTGAAGGACCCGATTACCGGTCGTAGCAGCGTTGAACTGCTGCCGACCTTTGATACCATCACCTATCGTGAGCTGCGTGAACGTGCGACCGCGATTGCGAGCGACCTGGCGCACCACCCGCAGGCGCCGGCGAAACCGGGTGATTTCCTGGCGAGCATCGGTTTTATTAGCGTGGACTACGTTGCGATCGATATTGCGGGCGTGTTTGCGGGTCTGACCGCGGTTCCGCTGCAAACCGGTGCGACCCTGGCGACCCTGACCGCGATCACCGCGGAAACCGCGCCGACCCTGTTTGCGGCGAGCATTGAACACCTGCCGACCGCGGTGGATGCGGTTCTGGCGACCCCGAGCGTGCGTCGTCTGCTGGTTTTTGATTATCGTGCGGGTAGCGACGAGGATCGTGAAGCGGTGGAGGCGGCGAAGCGTAAAATTGCGGATGCGGGCAGCAGCGTGCTGGTTGATGTGCTGGACGAGGTTATTGCGCGTGGTAAAAGCGCGCCGAAAGCGCCGCTGCCGCCGGCGACCGATGCGGGTGACGATAGCCTGAGCCTGCTGATCTATACCAGCGGTAGCACCGGCACCCCGAAGGGTGCGATGTATCCGGAGCGTAACGTGGCGCACTTCTGGGGTGGCGTTTGGGCTGCGGCGTTTGATGAAGATGCGGCGCCGCCGGTGCCGGCGATCAACATTACCTTCCTGCCGCTGAGCCACGTTGCGAGCCGTCTGAGCCTGATGCCGACCCTGGCGCGTGGTGGCCTGATGCACTTCGTGGCGAAGAGCGACCTGAGCACCCTGTTTGAGGATCTGAAACTGGCGCGTCCGACCAACCTGTTTCTGGTTCCGCGTGTGGTTGAGATGCTGTACCAGCACTATCAAAGCGAACTGGACCGTCGTGGCGTGCAGGATGGCACCCGTGAGGCGGAAGCGGTTAAAGATGATCTGCGTACCGGCCTGCTGGGTGGCCGTATCCTGACCGCGGGTTTTGGCAGCGCGCCGCTGAGCGCGGAGCTGGCGGGTTTTATCGAAAGCCTGCTGCAAATTCACCTGGTTGATGGTTATGGCAGCACCGAAGCGGGTCCGGTTTGGCGTGATGGTTACCTGGTGAAGCCGCCGGTTACCGACTATAAACTGATTGATGTGCCGGAGCTGGGCTATTTTAGCACCGACAGCCCGCACCCGCGTGGTGAACTGGCGATCAAGACCCAGACCATTCTGCCGGGTTACTATAAACGTCCGGAAACCACCGCGGAAGTGTTCGACGAAGATGGCTTTTACCTGACCGGTGACGTGGTTGCGCAGATCGGTCCGGAGCAATTCGCGTATGTGGATCGTCGTAAGAACGTTCTGAAACTGAGCCAGGGCGAGTTTGTGACCCTGGCGAAGCTGGAAGCGGCGTACAGCAGCAGCCCGCTGGTGCGTCAACTGTTCGTTTACGGTAGCAGCGAACGTAGCTATCTGCTGGCGGTTATTGTGCCGACCCCGGACGCGCTGAAGAAATTTGGTGTGGGTGAAGCGGCGAAGGCGGCGCTGGGTGAAAGCCTGCAGAAAATTGCGCGTGATGAGGGTCTGCAAAGCTATGAAGTGCCGCGTGACTTCATCATTGAAACCGACCCGTTTACCGTTGAAAACGGTCTGCTGAGCGACGCGCGTAAGAGCCTGCGTCCGAAGCTGAAAGAGCACTACGGCGAACGTCTGGAGGCGATGTATAAAGAACTGGCGGATGGTCAGGCGAACGAACTGCGTGATATCCGTCGTGGTGTGCAGCAACGTCCGACCCTGGAAACCGTTCGTCGTGCTGCGGCGGCGATGCTGGGTGCGAGCGCGGCGGAAATTAAGCCGGACGCGCACTTCACCGATCTGGGTGGCGACAGCCTGAGCGCGCTGACCTTCAGCAACTTTCTGCACGACCTGTTTGAGGTTGATGTGCCGGTTGGCGTGATCGTTAGCGCGGCGAACACCCTGGGTAGCGTGGCGGAACACATTGATGCGCAGCTGGCGGGTGGCCGTGCGCGTCCGACCTTCGCGACCGTTCACGGTAAAGGCAGCACCACCATCAAAGCGAGCGATCTGACCCTGGACAAATTTATTGATGAGCAAACCCTGGAAGCGGCGAAACACCTGCCGAAACCGGCGGACCCGCCGCGTACCGTTCTGCTGACCGGTGCGAACGGTTGGCTGGGTCGTTTCCTGGCGCTGGAATGGCTGGAGCGTCTGGCGCCGGCGGGTGGCAAGCTGATCACCATTGTTCGTGGCAAGGATGCGGCGCAGGCGAAAGCGCGTCTGGACGCGGCGTACGAGAGCGGCGATCCGAAACTGGCGGGTCACTATCAGGACCTGGCGGCGACCACCCTGGAAGTGCTGGCGGGTGACTTCAGCGAGCCGCGTCTGGGTCTGGATGAAGCGACCTGGAACCGTCTGGCGGATGAGGTTGACTTCATCAGCCACCCGGGTGCGCTGGTGAACCACGTTCTGCCGTACAACCAGCTGTTTGGCCCGAACGTGGCGGGTGTTGCGGAAATCATTAAGCTGGCGATCACCACCCGTATTAAACCGGTGACCTATCTGAGCACCGTTGCGGTTGCGGCGGGCGTTGAGCCGAGCGCGCTGGACGAAGATGGTGACATCCGTACCGTGAGCGCGGAGCGTAGCGTTGACGAAGGTTACGCGAACGGTTATGGCAACAGCAAATGGGGTGGCGAGGTGCTGCTGCGTGAGGCGCATGACCGTACCGGTCTGCCGGTGCGTGTTTTCCGTAGCGATATGATTCTGGCGCACCAGAAGTACACCGGCCAAGTGAACGCGACCGACCAGTTCACCCGTCTGGTTCAAAGCCTGCTGGCGACCGGTCTGGCGCCGAAGAGCTTCTACGAGCTGGATGCGCAGGGCAACCGTCAACGTGCGCACTATGACGGTATCCCGGTGGATTTTACCGCGGAGAGCATTACCACCCTGGGTGGCGACGGCCTGGAAGGTTACCGTAGCTATAACGTTTTCAACCCGCACCGTGATGGTGTGGGCCTGGACGAGTTTGTTGATTGGCTGATCGAAGCGGGCCACCCGATCACCCGTATTGACGATTACGACCAGTGGCTGAGCCGTTTCGAAACCAGCCTGCGTGGTCTGCCGGAAAGCAAGCGTCAAGCGAGCGTGCTGCCGCTGCTGCATGCGTTTGCGCGTCCGGGTCCGGCGGTTGATGGTAGCCCGTTCCGTAACACCGTGTTTCGTACCGACGTTCAGAAGGCGAAAATCGGCGCGGAACACGATATTCCGCACCTGGGCAAGGCGCTGGTTCTGAAATACGCGGACGATATCAAACAACTGGGTCTGCTGTAA |
| *TtCAR* | ATGACCGCGATGCGTCAGCGTACCGACTACGGCAAGCGTCTGCTGGTTAACATCGTGGATGAACGTGCGGAAACCGAACCGAACCGTGAGTGGGTGAGCATTCCGACCAGCAGCAACCCGAAAGACGGCTGGAAGAAAATCACCTATCGTCAAGCGGCGAACGCGGTTAACCGTGTGGCGCACAAGCTGGTTAGCAGCACCGGTCGTCCGAAAGAGGGCGAGTTCCCGACCGTTGCGTACATCGGTCCGAACGATGTGCGTTATGTGGTTTTCGCGCTGGGTGCGATTAAGGCGGGCTACCAGGCGCTGTTTATCAGCCCGCGTAACAGCCAGGAAGGCCAACTGAACCTGTTCGAGCTGACCAACTGCCGTACCATTTGGTTTGACGCGATGTATAAAGATGCGGTTCAGAGCTGGGTGCAAGAGCGTGACATGCACGCGATCATGACCTTCCCGGTTGCGGCGTGGTTTCCGGACGAGGATGTGGAACCGTACCCGTATGACAAGACCTTCGAGCAGGCGGAATGGGACCCGCTGATGGTGCTGCACACCAGCGGCAGCACCGGTTTTCCGAAACCGGTGGTTGTGCGTCAGGGTATGCTGGCGATTGGCGACGGTTACCACAACCTGGGTGAATGGAAGGGCCGTAAAATCTGGCTGGATGAGATGAGCCGTCGTAGCAAGCGTATGCTGTGCCCGATGCCGCTGTTCCATGCTGCGGCGATGTACGTTACCCTGCTGCTGGTGCAGTATTGGGATCTGCCGGTTGCGCTGGGCATTGGTGACCGTCCGCTGAGCGCGGATATGGCGCTGGAATGCCTGAAATACGCGGAAGTGGACAGCGTGATTCTGCCGCCGGCGATCCTGGAGGAACTGGGTCAGACCCAAGAAGCGATCGATGCGCTGAAGGCGCTGAGCTTCGTGGCGTTTGGTGGCGGTAACCTGAGCGACGAGGCGGGTGATAAGCTGGCGAAAGCGGGCGTTAAACTGTTCAACGCGATTAGCACCACCGAATTTGCGCCGTACCCGCTGTATTGGCAGACCAACCCGGAGCTGTGGCGTTACTTCATCTTTAACAGCGAACTGTTTGGTTGCGAGTGGCGTCCGGCGACCGACGAAAACACCTATGAGCAGGTTATTGTGCGTAAGGATAAACACCCGGGCTGCCAAGGTATCTTCTACACCTTTCCGGAGGCGTGGGAATATAGCACCAAGGACCTGTTCAGCCCGCACCCGACCCTGCCGGACCACTGGAAATACTGCGGTCGTAGCGATAACATCATTGTGTTTAGCAACGGCGAAAAGCTGAACCCGGCGAGCATTGAAACCATCCTGATGGGTCACCCGCGTGTGAAAGGTGCGCTGGTTGTGGGCAGCAACCGTTTCCAGCCGGCGCTGATTCTGGAGCCGGTTGAACACCCGCGTACCGAACAAGGCGTTCGTGAGTTTATCGACAGCGTGTGGCCGACCGTTGTGAAGGCGAACAAAGAAACCGTTGCGCACGGCCAGATTGGTCGTCAATTCATTGCGATCAGCAACCCGGATAAGCCGTTTCTGCGTGCGGGTAAAGGCACCATCCAGCGTGCGGGCACCATTCGTATCTACGAGGACGAAATTGATCAGATTTACGAACAAGCGGATGGTGTTGCGAGCAGCGAGGCGCCGGTGCTGAACCTGGAGAGCACCGAAAGCCTGACCCGTAGCATTGAAATCCTGTTCGAGAAGTGGCTGCAGGCGCCGAAACTGGAACCGGACACCGATTTCTTTACCGTTGGTATTGACAGCATGCAAGTGATCAACGCGAGCCGTCTGCTGCGTGCGGGCCTGGAGGCGGGCGGTGTTCGTGTGGATAGCGCGAGCCTGGCGACCCGTGTGATCTATGGTCACCCGACCGCGCGTCGTCTGGCGGAATACCTGTTCAGCGTTGTGAACCAGAAGGGTCAAGATGCGACCAGCGGTGAACCGCAGCATGAGGATCACGCGATGGAAGCGATCCTGGAGAAGTACACCCGTGACATGCCGCGTGCGCCGACCGCGGGTAAACCGGCGCCGGCGGATGAGGGCCAAGTTATCATTATTACCGGCACCACCGGCACCCTGGGCAGCTATACCCTGGACATTGCGAGCCGTTGCCCGCGTGTTCGTAAAGTGATCTGCCTGAACCGTAGCGACGATGCGGAAGCGCGTCAGCGTCGTAGCAACGCGGAGCGTGGTCTGCACATCGATTTCAGCAAGGCGGAATTTCTGCGTGCGGACATGAGCAAACACGATCTGGGCCTGGGTCGTGAGGCGTACGAACGTCTGCTGCACGAGGTTGACCGTGTGATTCACAACCAATGGCCGGTTAACTTCAACATGCCGGTGGAGAGCTTTGAACCGCACATCCGTGGTGTTCGTAACCTGGTGGATTTCAGCTGCAAGGCGACCAAACGTGTTCCGATTGTGTTTATCAGCAGCATTGCGACCGTTAACGGCTGGCGTAAGAGCGAAGCGGTGCCGGAGCGTAGCCTGAAAGAGCCGGAAATTGCGGTTGGCGGTTACGGTCGTAGCAAGCTGGTGAGCAGCCTGATCCTGGAAAAAGCGACCGAGGTTAGCGGCGTGCCGACCGAGATCATGCGTATTGGTCAGATCGCGGGCCCGAGCAGCGAAAAGGGTCTGTGGAACCGTCAAGAGTGGCTGCCGAGCATTATCGCGAGCAGCCTGTATCTGGGTGTGCTGCCGGACAGCCTGGGTCACATGAGCACCATTGATTGGACCCCGATTGAAGGTATCGCGAACCTGGTTCTGGAGGCGAGCGGCGTTACCAGCCAGGTGCCGCTGGAAGACATCAACGGTTACTTCCACGGCGTTAACCCGGTGACCACCCAGTGGCGTCCGCTGGCGGAAGCGGTGAAGGAGTTTTATGGCGGTCGTATTCAAAAACTGGTTCCGCTGGATGAGTGGGTGGAAGCGCTGGAGCAGAGCCAAGCGAAGGCGGAAGACATCAGCAAGAACCCGGGTGTTAAACTGCTGGATACCTATAAAGCGTGGGTGGCGGCGGCGCGTGAGGGTCAGAACTATGTTGCGATGGACACCAGCCGTACCACCAGCCGTAGCCGTACCATGCGTGAAATGCGTGCGATCACCCCGGAGCTGATGAAAAACTGGTGCCGTCAATGGGCGTTCTAA |
| *NcCAR* | ATGAGCCAGCAACAGAACCCGCCGTACGGTCGTCGTCTGATCCTGGACATCATTAAGGAACGTGCGCTGAACGAGCCGAACCGTGAATGGGTGAGCGTTCCGCGTAGCAGCGACCCGAAGGATGGCTGGAAAATCCTGACCTACCTGGATGCGTATAACGGTATTAACCGTGTGGCGCACAAACTGACCCAGGTTTGCGGTGCTGCGGCGCCGGGTAGCTTCCCGACCGTGGCGTACATCGGTCCGAACGACGTGCGTTATCTGGTTTTCGCGCTGGGCGCGGTTAAGGCGGGTTACAAAGCGCTGTTTATTAGCACCCGTAACAGCGCGGAGGCGCAAGTGAACCTGTTCGAACTGACCAACTGCAACGTGCTGGTTTTTGACCAAAGCTATAAAGCGACCGTTCAGCCGTGGCTGCATGAGCGTGAAATGACCGCGATCCTGGCGCTGCCGGCGGATGAGTGGTTTCCGGCGGACCAGGAAGATTTCCCGTACAACAAGACCTTTGAGGAAGCGGAGTGGGACCCGCTGATGGTGCTGCACACCAGCGGCAGCACCGGTTTCCCGAAACCGATCGTGGCGCGTCAAGGCATGCTGGCGGTTGCGGACCAGTTTCACAACCTGCCGCCGCGTGAAGATGGTAAACTGATGTGGATTGTTGAGATGAGCAAGCGTGCGAAACGTCTGATGCATCCGATGCCGCTGTTCCATGCGGCGGGCATGTACATCAGCATGCTGATGATTCACTATTGGGATACCCCGGGTGCGCTGGGTATTGGTGAACGTCCGCTGAGCAGCGACCTGGTGCTGGATTACATTGAGTATGCGGACGTTGAAGGCATGATCCTGCCGCCGGCGATTCTGGAGGAACTGAGCCGTGATGAGAAGGCGATCCAAAGCCTGCAGAAACTGAACTTCGTGAGCTTTGGTGGCGGTAACCTGGCGCCGGAGGCGGGTGACCGTCTGGTGGAAAACAACGTTACCCTGTGCAACCTGATTAGCGCGACCGAGTTCACCCCGTTCCCGTTTTACTGGCAATATGATCAGAAGCTGTGGCGTTACTTCAACTTTGACACCGACCTGTTCGGCATCGACTGGCGTCTGCACGATGGCGAGAGCACCTACGAACAAGTGATTGTTCGTAAGGATAAACACCCGGGCCTGCAGGGTTTCTTTTATACCTTCCCGGACAGCAGCGAATACAGCACCAAGGATCTGTATAAACGTCACCCGACCCACGAGGACTTCTGGATCTATCAGGGCCGTGCGGATAACATCATTGTTTTTAGCAACGGCGAGAAGCTGAACCCGATCACCATTGAGGAAACCCTGCAAGGCCACCCGAAAGTGATGGGCGCGGTGGTTGTGGGCACCAACCGTTTCCAGCCGGCGCTGATCATTGAGCCGGTTGAACACCCGGAAACCGAGGAAGGTCGTAAGGCGCTGCTGGACGAAATCTGGCCGACCGTTGTGCGTGTGAACAAAGAAACCGTTGCGCACGGCCAAATTGGTCGTCAGTACATGGCGCTGAGCACCCCGGGCAAGCCGTTTCTGCGTGCGGGTAAAGGCACCGTGCTGCGTCCGGGCACCATCAACATGTACAAGGCGGAAATCGACAAAATCTACGAAGATGCGGAGAAGGGCGTGGCGACCGATGAGGTTCCGAAGCTGGACCTGAGCAGCAGCGATGCGCTGATCGTTAGCATTGAGAAGCTGTTCGAAACCAGCCTGAACGCGCCGAAACTGGAAGCGGACACCGATTTCTTTACCGCGGGTGTGGACAGCATGCAAGTTATCACCGCGAGCCGTCTGATTCGTGCGGGCCTGGCGGCGGCGGGTGTGAACATTGAGGCGAGCGCGCTGGCGACCCGTGTTATTTATGGTAACCCGACCCCGAAGCGTCTGGCGGATTATCTGCTGAGCATCGTGAACAAAGACAGCAACCAGGGCACCCTGGACAACGAACACCACGTGATGGAGGCGCTGGTTGAAAAGTACACCCGTGACCTGCCGACCCCGAAGCAAAACAAGCCGGCGCCGGCGGATGAGGGTCAGGTTGTGGTTATCACCGGCACCACCGGCGGTATTGGTAGCTATCTGATCGACATCTGCAGCAGCAGCAGCCGTGTGAGCAAGATCATTTGCCTGAACCGTAGCGAAGACGGTAAAGCGCGTCAAACCGCGAGCAGCAGCGGTCGTGGTCTGAGCACCGATTTCAGCAAATGCGAGTTTTACCACGCGGACATGAGCCGTGCGGATCTGGGCCTGGGTCCGGAAGTTTATAGCCGTCTGCTGAGCGAGGTGGACCGTGTTATCCACAACCAGTGGCCGGTGAACTTCAACATCGCGGTTGAGAGCTTCGAGCCGCACATTCGTGGCTGCCGTAACCTGGTGGACTTCAGCTACAAGGCGGATAAAAACGTTCCGATCGTGTTTGTTAGCAGCATTGGCACCGTGGATCGTTGGCACGACGAGGATCGTATCGTTCCGGAAGCGAGCCTGGACGATCTGAGCCTGGCGGCGGGCGGTTATGGCCAAAGCAAGCTGGTGAGCAGCCTGATTTTTGACAAAGCGGCGGAAGTGAGCGGTGTGCCGACCGAAGTGGTTCGTGTGGGCCAAGTTGCGGGTCCGAGCAGCGAAAAGGGTTACTGGAACAAACAGGAATGGCTGCCGAGCATTGTGGCGAGCAGCGCGTATCTGGGCGTTCTGCCGGACAGCCTGGGTCAGATGACCACCATTGATTGGACCCCGATCGAAGCGATTGCGAAGCTGCTGCTGGAAGTGAGCGGTGTGATCGACAACGTGCCGCTGGATAAAATTAACGGCTACTTCCACGGTGTTAACCCGGAACGTACCAGCTGGAGCGCGCTGGCGCCGGCGGTGCAAGAGTACTATGGCGACCGTATCCAGAAGATTGTTCCGCTGGATGAATGGCTGGAGGCGCTGGAAAAGAGCCAAGAGAAAGCGGAAGACGTTACCCGTAACCCGGGTATCAAGCTGATTGATACCTACCGTACCTGGAGCGAGGGCTATAAGAAGGGCACCAAATTCGTGCCGCTGGACATGACCCGTACCAAGGAGTACAGCAAAACCATGCGTGAAATGCACGCGGTTACCCCGGAGCTGATGAAAAACTGGTGCCGTCAGTGGAACTTTTAA |
| *BsPPTase* | ATGAAGATTTACGGTATCTATATGGATCGTCCGCTGAGCCAGGAAGAGAACGAGCGTTTCATGACCTTTATTAGCCCGGAGAAGCGTGAAAAATGCCGTCGTTTCTACCACAAAGAAGACGCGCACCGTACCCTGCTGGGTGATGTGCTGGTTCGTAGCGTGATTAGCCGTCAGTACCAACTGGACAAGAGCGATATCCGTTTTAGCACCCAGGAGTATGGCAAACCGTGCATCCCGGACCTGCCGGATGCGCACTTCAACATTAGCCACAGCGGTCGTTGGGTTATCGGCGCGTTTGACAGCCAACCGATCGGCATTGATATCGAGAAGACCAAACCGATTAGCCTGGAAATCGCGAAGCGTTTCTTTAGCAAAACCGAGTATAGCGATCTGCTGGCGAAGGACAAAGATGAACAGACCGACTACTTCTATCACCTGTGGAGCATGAAGGAGAGCTTTATTAAGCAAGAAGGTAAAGGCCTGAGCCTGCCGCTGGACAGCTTCAGCGTGCGTCTGCACCAGGATGGTCAAGTTAGCATTGAGCTGCCGGACAGCCACAGCCCGTGCTACATCAAGACCTATGAAGTGGACCCGGGCTACAAAATGGCGGTTTGCGCGGCGCACCCGGACTTTCCGGAAGATATCACcATGGTGAGCTACGAGGAACTGCTGTAA |
| *NiPPTase* | ATGATCGAAACCATCCTGCCGGCGGGTGTGGAGAGCGCGGAACTGCTGGAGTATCCGGAAGACCTGAAAGCGCACCCGGCGGAGGAACACCTGATCGCGAAGAGCGTTGAGAAGCGTCGTCGTGATTTCATTGGTGCGCGTCACTGCGCGCGTCTGGCGCTGGCGGAGCTGGGTGAACCGCCGGTTGCGATTGGTAAAGGTGAACGTGGCGCGCCGATTTGGCCGCGTGGTGTGGTTGGCAGCCTGACCCATTGCGATGGTTACCGTGCTGCGGCGGTTGCGCACAAAATGCGTTTTCGTAGCATCGGTATTGATGCGGAGCCGCATGCGACCCTGCCGGAAGGCGTGCTGGATAGCGTTAGCCTGCCGCCGGAGCGTGAATGGCTGAAAACCACCGACAGCGCGCTGCACCTGGATCGTCTGCTGTTCTGCGCGAAAGAGGCGACCTATAAAGCGTGGTGGCCGCTGACCGCGCGTTGGCTGGGTTTCGAGGAAGCGCACATCACCTTTGAGATTGAAGACGGCAGCGCGGATAGCGGTAACGGCACCTTTCACAGCGAACTGCTGGTGCCGGGTCAGACCAACGATGGTGGCACCCCGCTGCTGAGCTTCGACGGTCGTTGGCTGATCGCGGATGGCTTTATCCTGACCGCGATTGCGTATGCGTAA |
| *TpPAL1* | ATGGAGGGTATCACCAACGGCCACGCGGAAGCGACCTTCTGCGTGACCAAGAGCGTTGGTGACCCGCTGAACTGGGGTGCGGCGGCGGAGAGCCTGACCGGTAGCCACCTGGATGAAGTGAAGCGTATGGTTGAGGAATACCGTAACCCGCTGGTGAAAATCGGTGGCGCGACCCTGACCATTGCGCAGGTTGCGGGTATTGCGAGCCATGACAGCGGCGTGCGTGTTGAGCTGAGCGAAAGCGCGCGTGCGGGTGTGAAAGCGAGCAGCGACTGGGTTATGGATAGCATGAACAACGGCACCGATAGCTACGGCGTTACCACCGGTTTTGGTGCGACCAGCCATCGTCGTACCAAGCAGGGTGGCGCGCTGCAAAAAGAGCTGATTCGTTTCCTGAACGCGGGCATCTTTGGTAACGGCACCGAAAGCAACTGCACCCTGCCGCATACCGCGACCCGTGCGGCGATGCTGGTTCGTATCAACACCCTGCTGCAAGGTTACAGCGGCATCCGTTTTGAGATTCTGGAAGCGATCACCAAGCTGCTGAACAACAACATTACCCCGTGCCTGCCGCTGCGTGGCACCATTACCGCGAGCGGCGACCTGGTGCCGCTGAGCTATATTGCGGGTCTGCTGACCGGCCGTCCGAACAGCAAGGCGGTTGGTCCGAGCGGCGAGATTCTGAACGCGAAAGAAGCGTTCCAGCTGGCGGGTATCGGCAGCGAGTTCTTTGAACTGCAACCGAAAGAGGGTCTGGCGCTGGTGAACGGCACCGCGGTTGGTAGCGGCCTGGCGAGCATTGTGCTGTTTGAGGCGAACGTGCTGGCGGTTCTGAGCGAAGTGATGAGCGCGATCTTCGCGGAAGTGATGCAGGGCAAGCCGGAATTTACCGACCACCTGACCCACAAGCTGAAACACCACCCGGGTCAAATCGAGGCTGCGGCGATTATGGAACACATCCTGGATGGCAGCGCGTACGTGAAAGCGGCGAAGAAACTGCACGAAACCGACCCGCTGCAGAAGCCGAAACAAGATCGTTATGCGCTGCGTACCAGCCCGCAGTGGCTGGGTCCGCTGATCGAAGTTATTCGTTTCAGCACCAAGAGCATTGAGCGTGAAATCAACAGCGTGAACGACAACCCGCTGATTGATGTTAGCCGTAACAAAGCGATCCACGGTGGCAACTTTCAGGGCACCCCGATTGGCGTGAGCATGGATAACACCCGTCTGGCGCTGGCGAGCATCGGCAAGCTGATGTTCGCGCAATTTAGCGAGCTGGTTAACGACTTCTACAACAACGGCCTGCCGAGCAACCTGACCGCGAGCCGTAACCCGAGCCTGGATTATGGTTTTAAAGGCGCGGAGATCGCGATGGCGAGCTACTGCAGCGAACTGCAGTATCTGGCGAACCCGGTGACCACCCACGTTCAAAGCGCGGAACAGCACAACCAAGACGTGAACAGCCTGGGTCTGATTAGCAGCCGTAAGACCAACGAGAGCATCGAAATTCTGAAACTGATGAGCAGCACCTTCCTGATTGCGCTGTGCCAGGCGATCGATCTGCGTCACCTGGAGGAAAACCTGCGTAACACCGTGAAGAACACCGTGAGCCAAGTTGCGAAACGTACCCTGACCACCGGTGTTAACGGCGAGCTGCACCCGAGCCGTTTCTGCGAAAAGGACCTGCTGAAAGTGGTTGATCGTGAGTACGTTTTTGCGTATGCGGATGATCCGTGCCTGGCGACCTACCCGCTGATGCAGAAGCTGCGTCAAGTGCTGGTTGACCACGCGCTGGTGAACGTTGATGGTGAAAAGAACAGCAACACCAGCATTTTCCAGAAAATCGCGACCTTTGAGGACGAACTGAAGGCGATCCTGCCGAAAGAGGTGGAAAGCACCCGTGTTGCGTATGAGAACGGTCAATGCGGCATTAGCAACAAGATCAAAGAATGCCGTAGCTACCCGCTGTATAAGTTCGTGCGTGAAGAACTGGGCACCGCGCTGCTGACCGGCGAGAAAGTTATTAGCCCGGGCGAGGAATGCGACAAGCTGTTTACCGCGATGTGCCAGGGCAAAATCGTTGATCCGCTGCTGGAGTGCATGGGTGAATGGAACGGCGCGCCGCTGCCGATTTGCTAA |
| *AtPAL2* | ATGGACCAGATCGAAGCGATGCTGTGCGGTGGCGGTGAGAAGACCAAAGTGGCGGTTACCACCAAAACCCTGGCGGACCCGCTGAACTGGGGCCTGGCGGCGGACCAGATGAAGGGTAGCCACCTGGATGAAGTGAAGAAAATGGTTGAGGAATACCGTCGTCCGGTGGTTAACCTGGGTGGTGAAACCCTGACCATTGGCCAAGTGGCGGCGATCAGCACCGTTGGCGGTAGCGTGAAGGTTGAACTGGCGGAAACCAGCCGTGCGGGTGTGAAAGCGAGCAGCGACTGGGTTATGGAGAGCATGAACAAGGGCACCGATAGCTATGGTGTTACCACCGGTTTTGGTGCGACCAGCCACCGTCGTACCAAAAACGGCACCGCGCTGCAAACCGAACTGATTCGTTTCCTGAACGCGGGCATCTTTGGTAACACCAAGGAAACCTGCCATACCCTGCCGCAGAGCGCGACCCGTGCGGCGATGCTGGTGCGTGTTAACACCCTGCTGCAAGGCTACAGCGGTATCCGTTTTGAAATTCTGGAGGCGATCACCAGCCTGCTGAACCACAACATTAGCCCGAGCCTGCCGCTGCGTGGCACCATTACCGCGAGCGGTGACCTGGTGCCGCTGAGCTATATTGCGGGCCTGCTGACCGGTCGTCCGAACAGCAAAGCGACCGGTCCGGATGGTGAAAGCCTGACCGCGAAGGAAGCGTTCGAGAAAGCGGGCATCAGCACCGGTTTCTTTGATCTGCAGCCGAAAGAGGGTCTGGCGCTGGTGAACGGCACCGCGGTTGGCAGCGGTATGGCGAGCATGGTTCTGTTTGAAGCGAACGTGCAAGCGGTTCTGGCGGAAGTGCTGAGCGCGATTTTCGCGGAAGTTATGAGCGGTAAACCGGAGTTTACCGACCACCTGACCCACCGTCTGAAACATCACCCGGGTCAGATTGAAGCTGCGGCGATTATGGAGCACATCCTGGATGGTAGCAGCTACATGAAGCTGGCGCAAAAAGTGCACGAAATGGACCCGCTGCAGAAGCCGAAACAAGATCGTTATGCGCTGCGTACCAGCCCGCAGTGGCTGGGTCCGCAAATCGAGGTTATTCGTCAGGCGACCAAAAGCATTGAACGTGAGATCAACAGCGTGAACGACAACCCGCTGATTGATGTTAGCCGTAACAAGGCGATCCACGGCGGTAACTTCCAGGGCACCCCGATTGGTGTGAGCATGGATAACACCCGTCTGGCGATCGCGGCGATTGGCAAACTGATGTTCGCGCAATTTAGCGAACTGGTTAACGACTTCTACAACAACGGTCTGCCGAGCAACCTGACCGCGAGCAGCAACCCGAGCCTGGATTATGGCTTTAAGGGTGCGGAAATCGCGATGGCGAGCTACTGCAGCGAGCTGCAGTATCTGGCGAACCCGGTGACCAGCCACGTTCAAAGCGCGGAACAGCACAACCAAGACGTGAACAGCCTGGGCCTGATTAGCAGCCGTAAGACCAGCGAGGCGGTTGATATCCTGAAACTGATGAGCACCACCTTTCTGGTGGGTATTTGCCAGGCGGTTGACCTGCGTCACCTGGAGGAAAACCTGCGTCAGACCGTGAAAAACACCGTGAGCCAAGTTGCGAAGAAAGTTCTGACCACCGGCATCAACGGTGAACTGCACCCGAGCCGTTTCTGCGAGAAGGACCTGCTGAAAGTGGTTGATCGTGAGCAGGTGTTTACCTACGTTGACGATCCGTGCAGCGCGACCTATCCGCTGATGCAGCGTCTGCGTCAAGTGATCGTTGATCACGCGCTGAGCAACGGCGAAACCGAGAAGAACGCGGTTACCAGCATTTTCCAAAAAATCGGTGCGTTTGAGGAAGAGCTGAAGGCGGTGCTGCCGAAAGAAGTTGAAGCGGCGCGTGCGGCGTACGGCAACGGCACCGCGCCGATTCCGAACCGTATCAAAGAATGCCGTAGCTACCCGCTGTATCGTTTCGTGCGTGAAGAGCTGGGCACCAAGCTGCTGACCGGCGAGAAAGTGGTTAGCCCGGGCGAAGAGTTCGACAAGGTTTTTACCGCGATGTGCGAAGGTAAACTGATCGACCCGCTGATGGATTGCCTGAAGGAGTGGAACGGTGCGCCGATCCCGATTTGCTAA |
| *ZmPAL2* | ATGGAGTGCGAAAACGGTCGTGGCGTTGCGGCGACCAACAGCGACAGCCTGTGCATGGCGACCCCGCGTGCGGACCCGCTGAACTGGGGCAAGGCGGCGGAGGAACTGATGGGCAGCCACCTGGACGAGGTGAAGCGTATGGTTGCGGAATACCGTCAGCCGCTGGTTAAAATTGAGGGTGCGAGCCTGAGCATTGCGCAAGTTGCGGCGGTTGCGACCGGTGCGGGTGAAGCGCGTGTGGAACTGGATGAGAGCGCGCGTAGCCGTGTTAAGGCGAGCAGCGACTGGGTGATGACCAGCATGATGAACGGCACCGATAGCTATGGCGTTACCACCGGTTTTGGTGCGACCAGCCACCGTCGTACCAAAGAGGGTGGCGCGCTGCAGCGTGAACTGATTCGTTTCCTGAACGCGGGTGCGTTTGGCACCGGTGCGGATGGTCATGTTCTGCCGGCGGAAACCACCCGTGCGGCGATGCTGGTGCGTATCAACACCCTGCTGCAAGGTTACAGCGGCATCCGTTTTGAAATTCTGGAGGCGATCGTTAAGCTGCTGAACGCGAACGTGACCCCGTGCCTGCCGCTGCGTGGCACCGTTACCGCGAGCGGCGACCTGGTGCCGCTGAGCTATATTGCGGGTCTGGTTACCGGTCGTGAGAACAGCGTGGCGGTTGCGCCGGATGGTAGCAAGGTGAACGCGGCGGAAGCGTTCAAAATCGCGGGCATTCAGGGTGGCTTCTTTGAGCTGCAACCGAAAGAAGGTCTGGCGATGGTTAACGGCACCGCGGTGGGTAGCGGCCTGGCGAGCACCGTTCTGTTCGAGGCGAACATCCTGGCGATTCTGGCGGAAGTGCTGAGCGCGGTGTTCTGCGAGGTGATGAACGGCAAGCCGGAATACACCGACCACCTGACCCACAAGCTGAAACATCATCCGGGTCAGATTGAGGCTGCGGCGATTATGGAACACATCCTGGAGGGTAGCAGCTACATGAAACTGGCGAAGAAACTGGGCGAACTGGACCCGCTGATGAAGCCGAAACAAGATCGTTATGCGCTGCGTACCAGCCCGCAGTGGCTGGGTCCGCAAATCGAAGTGATTCGTGCGGCGACCAAGAGCATTGAGCGTGAAATCAACAGCGTTAACGACAACCCGCTGATTGATGTGGCGCGTAGCAAAGCGCTGCACGGTGGCAACTTCCAGGGCACCCCGATCGGCGTTAGCATGGATAACACCCGTCTGGCGATCGCGGCGATTGGCAAGCTGATGTTCGCGCAATTTAGCGAGCTGGTGAACGACTACTATAACAACGGCCTGCCGAGCAACCTGAGCGGTGGCCGTAACCCGAGCCTGGATTACGGTTTCAAAGGCGCGGAGATTGCGATGGCGAGCTATTGCAGCGAACTGCAGTTTCTGGGTAACCCGGTTACCAACCACGTGCAAAGCGCGGAACAGCACAACCAAGACGTTAACAGCCTGGGCCTGATCAGCAGCCGTAAGACCGCGGAAGCGATCGAGATTCTGAAACTGATGAGCAGCACCTTTCTGATTGCGCTGTGCCAGGCGGTGGATCTGCGTCACATCGAGGAAAACGTTAAGAGCGCGGTGAAAAGCTGCGTGATGACCGTTGCGAAGAAAACCCTGAGCACCAACAGCACCGGTGGCCTGCACGTTGCGCGTTTCTGCGAGAAAGACCTGCTGCAAGAGATCGAACGTGAGGCGGTGTTTGCGTACGCGGACGATCCGTGCAGCGCGAACTATCCGCTGATGAAGAAACTGCGTAACGTGCTGGTTGAACGTGCGCTGGCGAACGGTGCGGCGGAGTTCAACGCGGAAACCAGCGTGTTCGCGAAGGTGGCGCAGTTTGAGGAAGACCTGCGTGCGGCGCTGCCGAAAGCGGTTGAAGCGGCGCGTGCGGCGGTGGAAAACGGCACCGCGGGCATTCCGAACCGTATCGCGGAGTGCCGTAGCTACCCGCTGTATCGTTTTGTTCGTGAGGAACTGGGTGCGGTGTACCTGACCGGCGAAAAGACCCGTAGCCCGGGCGAGGAACTGAACAAGGTGCTGGTTGCGATTAACCAGGGCAAACACATCGATCCGCTGCTGGAATGCCTGAAAGAGTGGAACGGTGAACCGCTGCCGATCTGCTAA |
| *RgTAL* | ATGGCGCCGCGCCCGACTTCTCAAAGCCAGGCCCGCACTTGCCCGACCACCCAGGTTACCCAAGTTGATATCGTTGAGAAAATGCTGGCGGCTCCGACTGATAGCACCCTGGAGCTGGACGGTTATAGCCTGAACCTGGGTGATGTTGTGAGCGCTGCGCGTAAGGGTCGTCCGGTTCGTGTTAAAGATAGCGATGAAATCCGCAGCAAAATCGACAAGAGCGTTGAATTTCTGCGCAGCCAACTGAGCATGTCTGTTTACGGCGTGACTACCGGCTTCGGCGGCAGCGCGGACACCCGCACCGAGGACGCAATTAGCCTGCAAAAGGCGCTGCTGGAACACCAGCTGTGTGGTGTGCTGCCGTCGAGCTTTGACAGCTTCCGCCTGGGCCGTGGTCTGGAGAACAGCCTGCCGCTGGAAGTTGTTCGCGGTGCAATGACCATTCGTGTGAACTCTCTGACCCGTGGCCATAGCGCTGTTCGTCTGGTTGTTCTGGAAGCACTGACCAACTTTCTGAACCACGGTATTACCCCGATTGTTCCGCTGCGCGGTACCATCTCCGCGAGCGGCGATCTGTCTCCACTGTCTTACATTGCAGCGGCGATTAGCGGTCACCCGGATAGCAAAGTTCACGTGGTTCATGAAGGCAAAGAGAAGATCCTGTACGCGCGTGAAGCGATGGCGCTGTTCAACCTGGAACCGGTGGTTCTGGGTCCGAAGGAGGGCCTGGGTCTGGTGAACGGTACCGCAGTTTCCGCGAGCATGGCAACCCTGGCACTGCACGACGCGCACATGCTGAGCCTGCTGAGCCAATCTCTGACCGCGATGACCGTGGAGGCGATGGTTGGTCACGCGGGCAGCTTCCATCCATTCCTGCACGATGTTACCCGTCCGCACCCGACCCAAATCGAGGTTGCGGGTAACATTCGCAAACTGCTGGAGGGCTCTCGCTTCGCGGTTCACCACGAGGAAGAGGTTAAGGTTAAGGATGATGAAGGCATTCTGCGTCAGGATCGTTATCCGCTGCGCACCAGCCCGCAATGGCTGGGTCCGCTGGTGTCCGACCTGATTCACGCTCATGCCGTTCTGACCATCGAAGCGGGTCAAAGCACCACCGATAACCCACTGATCGATGTTGAGAACAAGACCAGCCATCATGGCGGCAACTTCCAGGCGGCAGCGGTTGCCAACACTATGGAAAAGACCCGTCTGGGCCTGGCCCAAATCGGTAAACTGAACTTCACCCAGCTGACCGAGATGCTGAACGCGGGCATGAACCGTGGCCTGCCGAGCTGCCTGGCGGCTGAAGACCCATCCCTGAGCTATCATTGCAAAGGTCTGGACATTGCGGCGGCTGCATATACCAGCGAACTGGGCCACCTGGCTAACCCGGTTACCACCCACGTTCAACCGGCTGAAATGGCAAACCAGGCGGTGAACAGCCTGGCGCTGATTAGCGCACGTCGTACCACCGAATCTAACGACGTTCTGTCCCTGCTGCTGGCAACCCACCTGTACTGCGTGCTGCAGGCGATCGACCTGCGTGCGATTGAGTTCGAGTTCAAGAAACAGTTTGGTCCGGCCATTGTTAGCCTGATCGACCAACACTTTGGTAGCGCGATGACCGGTAGCAACCTGCGTGATGAGCTGGTTGAAAAGGTTAACAAGACTCTGGCCAAGCGTCTGGAGCAAACCAACAGCTACGATCTGGTTCCGCGCTGGCATGACGCTTTCAGCTTCGCGGCAGGCACTGTTGTTGAGGTTCTGTCCAGCACCAGCCTGAGCCTGGCGGCCGTGAACGCATGGAAGGTTGCGGCAGCCGAGAGCGCGATCTCCCTGACCCGCCAGGTACGTGAAACCTTCTGGAGCGCTGCAAGCACCTCCAGCCCGGCGCTGTCTTACCTGAGCCCGCGCACCCAGATCCTGTACGCATTTGTGCGTGAGGAACTGGGTGTTAAAGCCCGCCGTGGTGACGTTTTCCTGGGTAAACAAGAAGTTACCATCGGCAGCAACGTTAGCAAGATTTACGAAGCCATCAAGAGCGGCCGTATCAACAACGTTCTGCTGAAGATGCTGGCATAA |
| *SriPAL* | ATGCACACcATGGACACCGCGCTGGCGGCGAACGATAAGGCGGAGCTGCTGATCGACGGCCACACCCTGACCGTTGCGGATGTGGTTAGCGGTGCGCGTCCGGCGGACACCACCCGTGTGCGTGCGCGTCTGGCGGAGGGTGCGGTTCAGCGTATCGAACAAAGCCTGGCGCTGAAGAACAAAGTTATTGAGGCGGGCCTGCCGGTGTACGGTGTGACCAGCGGTTTCGGCGATAGCAACACCCGTCAGATTAGCGGCCTGAAAAGCGAAGCGCTGCAAACCAACCTGATCCGTTTTCTGAGCTGCGGTATTGGTCCGGTGGCGACCCCGGATGTTATCCGTGCGACCATGATTGTTCGTGCGAACTGCCTGGCGCGTGGTGCGAGCGGCATCCGTACCGAGATTCTGGAACTGCTGCTGGACTGCCTGAACAACGATGTGCTGCCGCCGATTCCGGAACGTGGTAGCGTTGGTGCGAGCGGTGATCTGGTGCCGCTGAGCTATGTTGCGGCGCTGCTGACCGGTCAGGGCAAGGCGCTGCACCAAGGCGAGGAAAAAGATGCGAGCGCGGCGCTGGCGGATGCGGGTCTGGGTGCGGTGGTTCTGGGTGCGAAAGAGGGTCTGGCGCTGGTGAACGGCACCAGCTTCATGAGCGGTTTTGCGACCCTGGCGGTTCACGATGCGACCGAACTGGCGTTTGCGGCGGACCTGAGCACCGCGCTGGCGAGCCAGGTGCTGCAAGGTAACCCGGGTCACTTTGTTCCGTTCATCTTTGACCAGAAGCCGCACACCGGCACCCGTACCAGCGCGCGTACCATTCGTGAGCTGCTGGGTAACCCGGAAGACTGCGATCCGAGCGTGGACCCGGAAGGTGCTGCGCTGACCGAAAGCGGTTTCCGTCAGCTGGAGGAACCGATCCAAGACCGTTATAGCGTTCGTTGCGCGCCGCACGTGACCGGTGTTCTGCGTGACACCCTGGATTGGGCGAAGAACTGGGTTGAGGTGGAAATTAACAGCACCAACGACAACCCGCTGTTCGATGTGGAGGCGGGCATGGTTCGTAACGGTGGCAACTTTTATGGTGGCCACGTGGGTCAGGCGATGGATGCGCTGAAGACCGCGGTGGCGAGCGTTGGCGACCTGCTGGATCGTCAACTGGAGCTGATCGTTGACGAAAAATTCAACAACGGTCTGACCCCGAACCTGATTCCGCGTTTTGACGCGGATAGCTGGGAAGCGGGTCTGCACCACGGCTTCAAGGGTATGCAGATTGCGGCGAGCGGCCTGACCGCGGAGGCGCTGAAAAACACCATGCCGGCGACCAGCTTTAGCCGTAGCACCGAAGCGCACAACCAGGATAAAGTTAGCATGGCGACCATTGCGGCGCGTGACGCGCGTACCGTGGTTGAGCTGGTGCGTCAAGTTGCGGCGATTCACCTGCTGGCGCTGTGCCAGGCGGCGGATCTGCGTGGTCAAGAATGCCTGAGCGCGCCGACCCGTGCGGCGTACGAACTGATTCGTAGCGTGAGCGCGACcATGGACGGCGATCGTCCGCTGGCGCGTGACATCGAACTGGTGGTTGGCCTGATTGCGAGCGGCGAGCTGCGTCGTGCGGTTGAAGACGCGGGTCGTGATTAA |

**
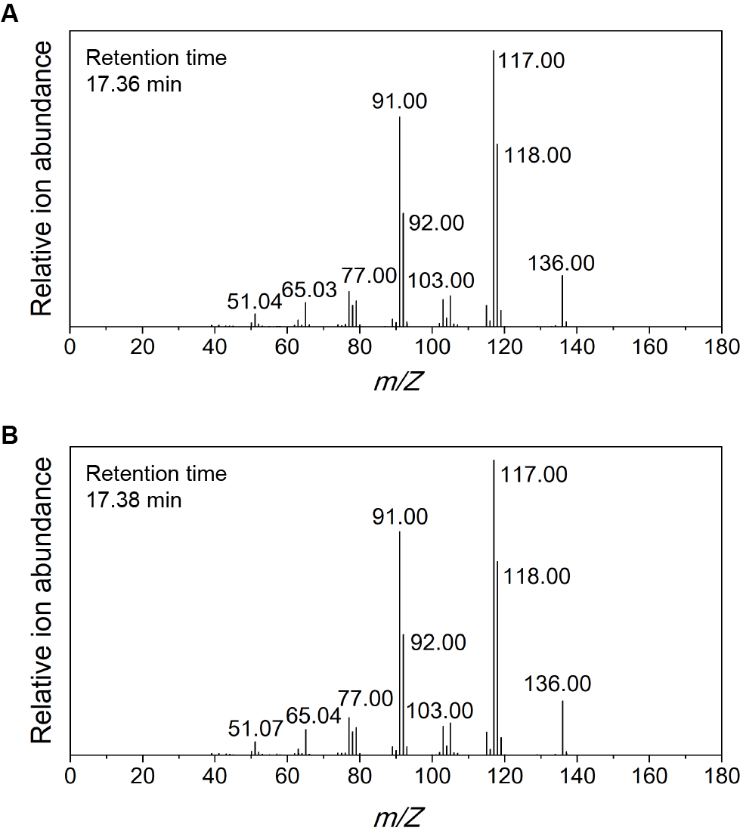
**

**Fig. S1:** GC-MS analysis for identification of 3-phenylpropanol. (A) Mass spectra of 3-phenylpropanol standard. (B) GC-MS analysis for culture supernatants of strain BTR01 at 48 h.
